# Design, synthesis and cytotoxic evaluation of novel bis-thiazole derivatives as preferential Pim1 kinase inhibitors with *in vivo* and *in silico* study

**DOI:** 10.1080/14756366.2023.2166936

**Published:** 2023-02-02

**Authors:** Mohammad M. Al-Sanea, Tamer M. Nasr, Samir Bondock, Aya Y. Gawish, Nada M. Mohamed

**Affiliations:** a Department of Pharmaceutical Chemistry, College of Pharmacy, Jouf University, Sakaka, Saudi Arabia; b Department of Pharmaceutical Chemistry, Faculty of Pharmacy, Modern University for Technology and Information (MTI) University, Cairo, Egypt; c Department of Pharmaceutical Chemistry, Faculty of Pharmacy, Helwan University, Helwan, Egypt; d Chemistry Department, Faculty of Science, King Khalid University, Abha, Saudi Arabia; e Chemistry Department, Faculty of Science, Mansoura University, Mansoura, Egypt; f Department of Pharmacology and Toxicology, Faculty of Pharmacy, Modern University for Technology and Information (MTI) University, Cairo, Egypt

**Keywords:** Pim1 inhibitor, bis-thiazole, cytotoxicity, *in silico* and *in vivo* study

## Abstract

Bis-thiazole derivatives were synthesised conforming to the Pim1 pharmacophore model following Hantzsch condensation. Pim1 has a major role in regulating the G1/S phase which upon inhibition the cell cycle stops at its early stages. Derivatives **3b** and **8b** showed the best Pim1 IC_50_ 0.32 and 0.24 µM, respectively relative to staurosporine IC_50_ 0.36 µM. Further confirmation of **3b** and **8b** Pim1 inhibition was implemented by hindering the T47D cell cycle at G0/G1 and S phases where **3b** showed 66.5% cells accumulation at G0/G1 phase while **8b** demonstrated 26.5% cells accumulation at the S phase compared to 53.9% and 14.9% of a control group for both phases, respectively. Additional *in vivo* cytotoxic evaluation of **3b** and **8b** revealed strong antitumor activity with up-regulation of caspase-3 and down-regulation of VEGF and TNF *α* immune expression with concomitant elevation of malondialdehyde levels in case of **8b**.

## Introduction

The World Health Organisation stated that over 19 million new cancer cases were confirmed in 2020 of which breast cancer claimed to be the top of all cancer types displaying over two million new cases followed by lung cancer^1^. Cancer aetiology and pathogenesis involve several enzymatic machineries, DNA mutation, and other predisposing factors therefore, it can arise from any tissue cell at any time irrespective of age or health state with the ability of metastasis^2^. Traditional chemotherapy shows multiple limitations and possible drug resistance among other factors that necessitate continuous study and development of new anticancer agents with better selectivity and safety^3^.

**Figure 1.**
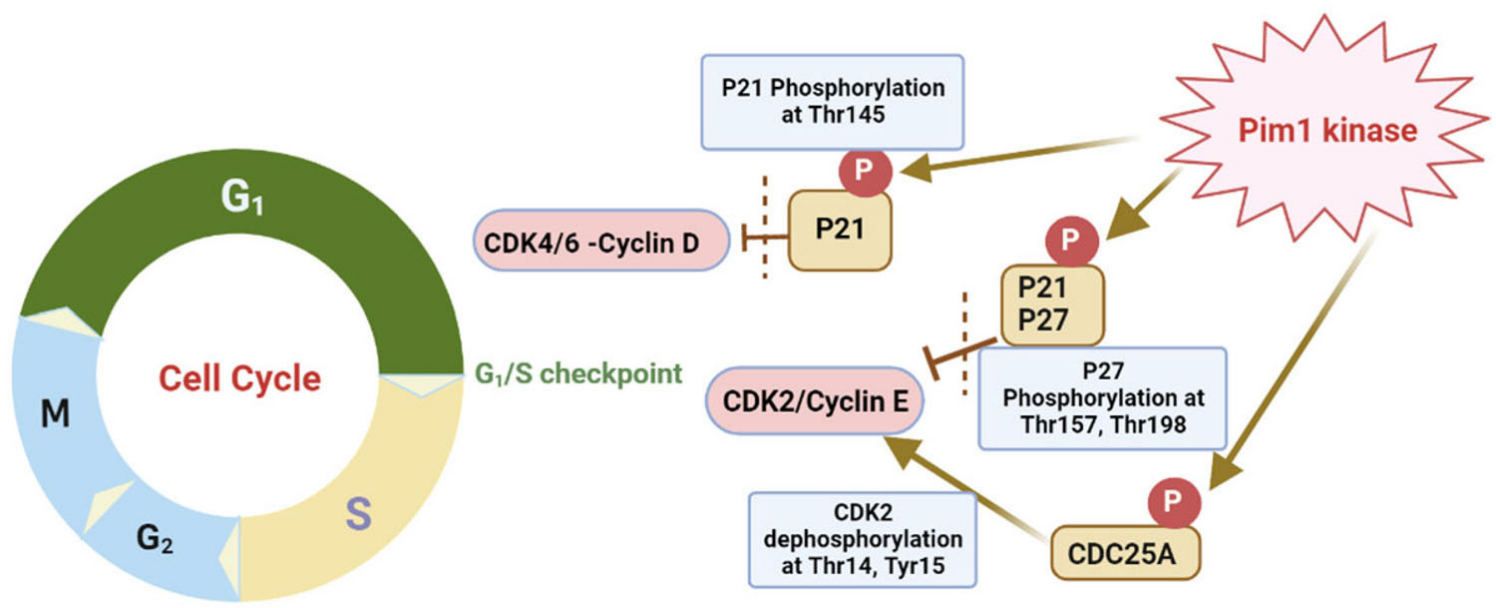
Graphical illustration of Pim1 kinase role during the cell cycle G1/S phase.

Cancerous cell is characterised by fast and continuous mitosis which is accompanied by irregularities in the normal cell cycle. Therefore, manipulating cell cycle regulating enzymes and proteins has been a major strategy in triggering apoptosis and cancerous cell death. One of the main regulating kinase enzymes of the cell cycle is Provirus Integration Site for Moloney Murine Leukaemia Virus (Pim). Pim enzymes are serine/threonine kinases with three isoforms Pim1, Pim2 and Pim3 out of which Pim1 is overexpressed in breast cancer, especially the triple negative type^4^^,^^5^ and has a significant role in prostate cancer progression^6^. Moreover, Pim1 inhibitors showed significant anticancer effect against HER2 positive cancer cells through HER2 down-regulation^7^. Overexpression of Pim1 enhanced the cell proliferation induced by mitogens or cytokins while its down-regulation was correlated with cell cycle regression^8^. Mechanistically, Pim1 kinase affects several cell cycle regulators such as the tumour suppressor proteins P21^Cip1^/^Waf1^, P27^Kip1^ and CDC25A (Figure 1). Pim1 inactivates both tumour suppressor proteins through phosphorylation of P21^Cip1^/^Waf1^ at Thr145 while for P27^Kip1^ occurs at Thr157 and Thr198^9^^,^^10^. Moreover, P27^Kip1^ phosphorylation by Pim1 leads to its degradation and exclusion from the cell nucleus^11^. Both proteins inactivation caused enhancement of CDKs binding to cyclin family hence, promotes cell proliferation specifically at G1/S phase. Consequently, inhibition of Pim1 kinase caused activation of those tumour suppressor proteins and ultimately stops the cell cycle at G1/S phase. Furthermore, Pim1 inhibition inactivates CDC25A phosphatase which in turn deactivates primarily CDK2 at G1/S phase by dephosphorylation at Thr14 and Tyr15 that supported the inhibition capability of Pim1 inhibitors to stop the cell cycle at the early phase^12^. Additionally, Pim1 inhibition was proved to assist in drug resistance cancers by inhibition of the consequent phosphorylation and activation of major causing drug-resistant proteins. Examples of those affected proteins are P-glycoprotein^13^, breast cancer resistant proteins (BCRP)^14^, and fms-like tyrosine kinase-3 (FLT-3)^15^. Another reason of being promising anticancer target is that Pim1 kinase is constitutively active with a non-significant conformational change between its apoprotein and bound conformations which suggested equal inhibition of both forms^16^.

**Figure 2.**
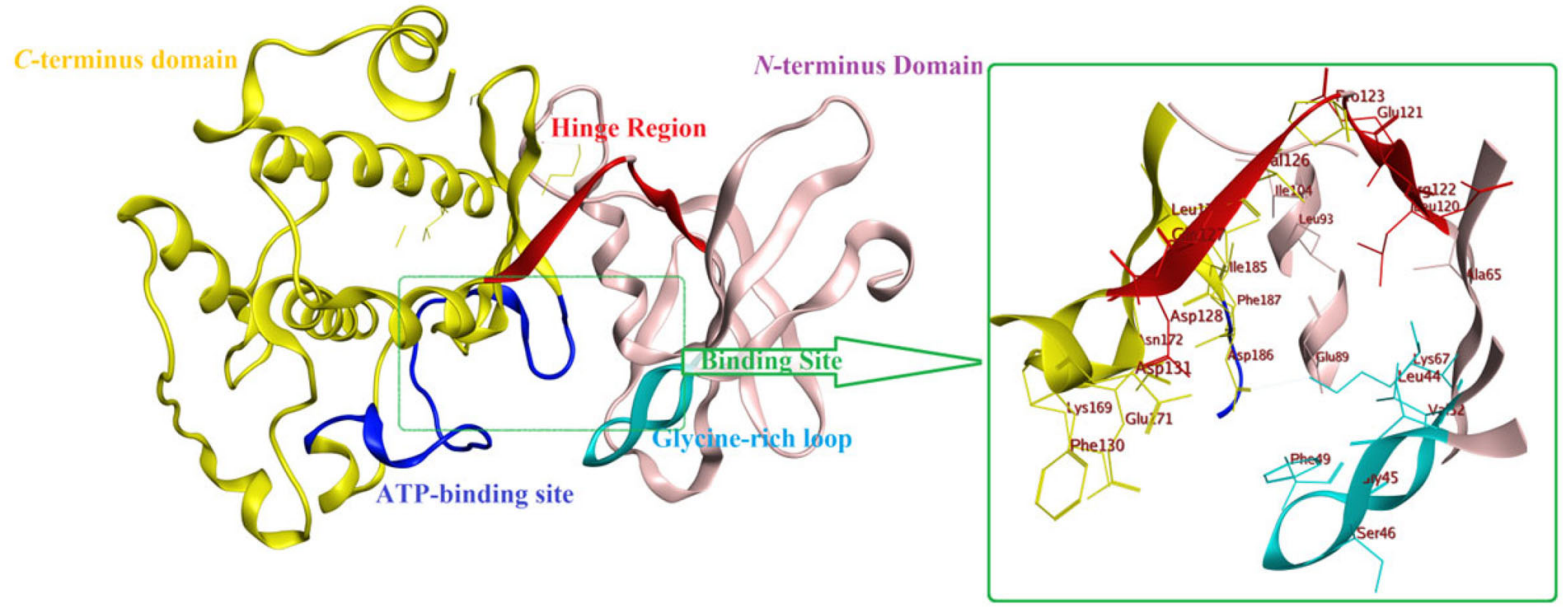
The protein crystal structure of Pim1 kinase (PDB ID: 4DTK) with a focus on the binding site residues.

The reported crystal structure of Pim1 enzyme possesses the common kinase features of having *N*-terminal (NTD) and *C*-terminal (CTD) domains connected by the hinge region where the ATP-binding site is located (Figure 2). Yet, its hinge region is unique in having only one hydrogen-bond donor to bind with the adenine nitrogen of ATP substrate. Structurally, Pim1 NTD comprises mainly of *β*-strands with one *α*-helix while its CTD is mainly *α-*helix (Figure 2) with its active site is located at the interface between those two domains. The hinge region, glycine-rich and the activation loop form a large umbrella to cover the groove-shaped active site^17^.

It is worth mentioning that Pim1 shared 55% sequence similarity with its analogue enzyme Pim2 with Val126 and Ser46 were replaced by Ala122 and Lys40, respectively, which accounted for preferential Pim1 inhibitor design^18^.

**Figure 3.**
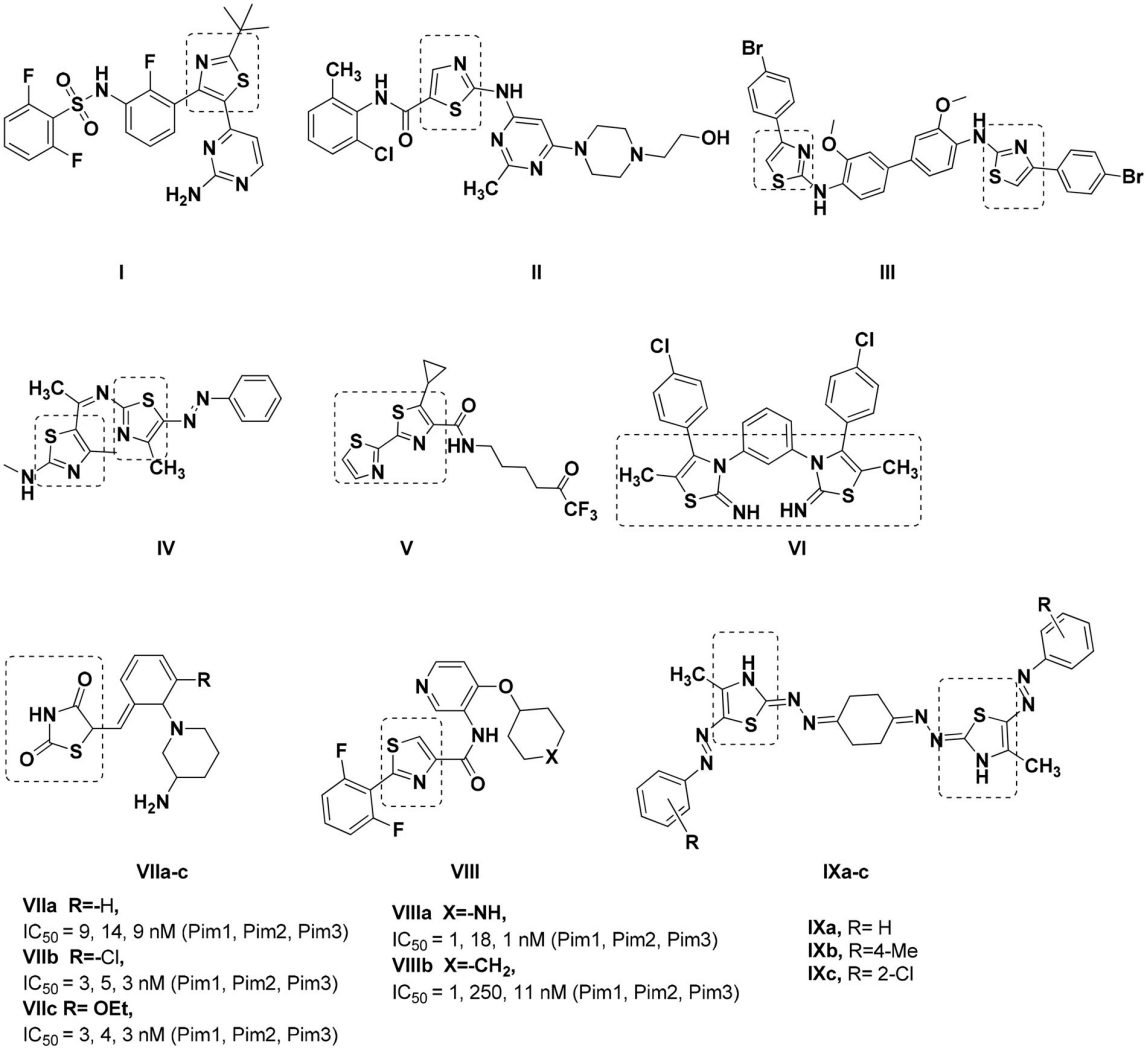
Reported thiazole, thiazolidinedione and bis-thiazole derivatives with anticancer activity.

Thiazole ring have been acknowledged as a promising scaffold for many anticancer drugs such as dabrafenib **I**^19^ and dasatinib **II**^20^ (Figure 3). Similarly, many bis-thiazole derivatives **III–VI** had established antitumor activity with IC_50_ of 15.69 µM against rat glioma, 13.24 µM, 20.81 nM against HepG2 and 1.46 µM against leukaemia, respectively^21–24^. Among the proven mechanisms of bis-thiazole derivatives is their ability to inhibit Pim1 kinase activity leading to cytotoxicity^25^. Thiazolidinedione **VIIa–c**^26^ and thiazole **VIIIa–b**^27^ analogues showed nanomolar Pim1 inhibitory activity despite being non-selective to Pim1 and showed appreciable inhibition of Pim2 and Pim3 as well (Figure 3). On the other hand, **IXa–c** quantitative RT-PCR analysis to quantify its corresponding mRNA expression of the pro-apoptotic *Pim1* gene showed 0.4-, 0.5-, 0.3-fold of change in Pim1 kinase activity, respectively^25^. Consequently, the reduction of Pim1 pro-apoptotic gene expression by **Ixa–c** was translated into decrease in its overall kinase activity by one third to half its original value.

**Figure 4.**
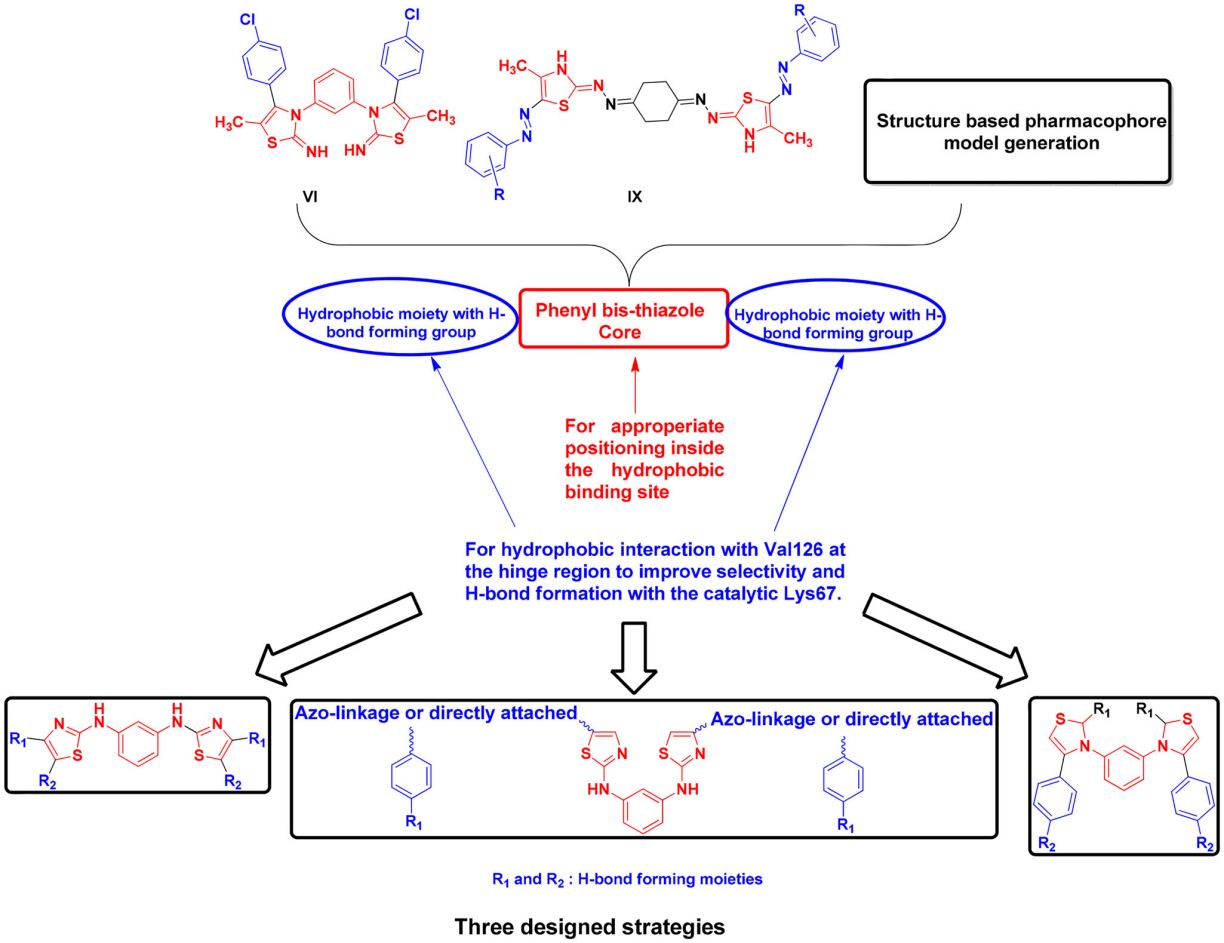
Schematic presentation of the study rationale.

**Figure 5.**
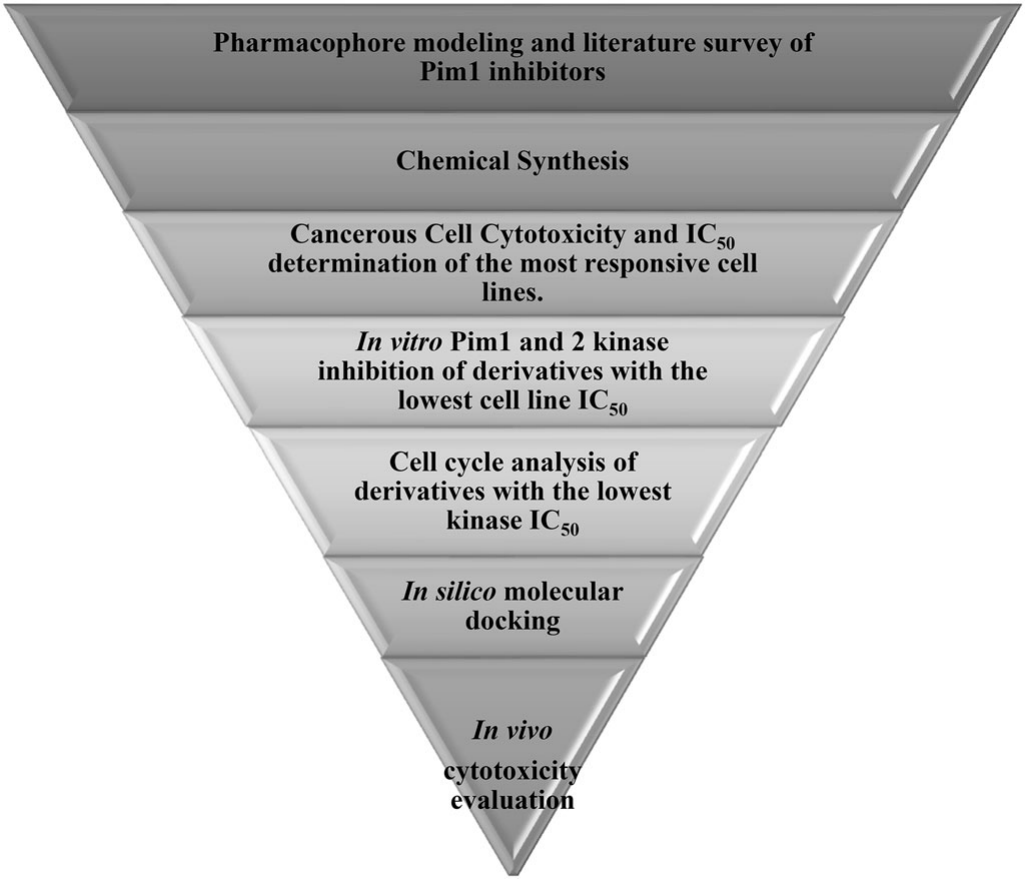
Schematic presentation of the study workflow.

Based on the earlier data of Pim1 kinase protein structure and the importance of bis-thiazole potential cytotoxicity, this study aimed to design novel bis-thiazole derivatives as preferential Pim1 inhibitors to treat the underlying cancer types. This study rational and workflow were summarised in Figures 4 and 5.

**Figure 6.**
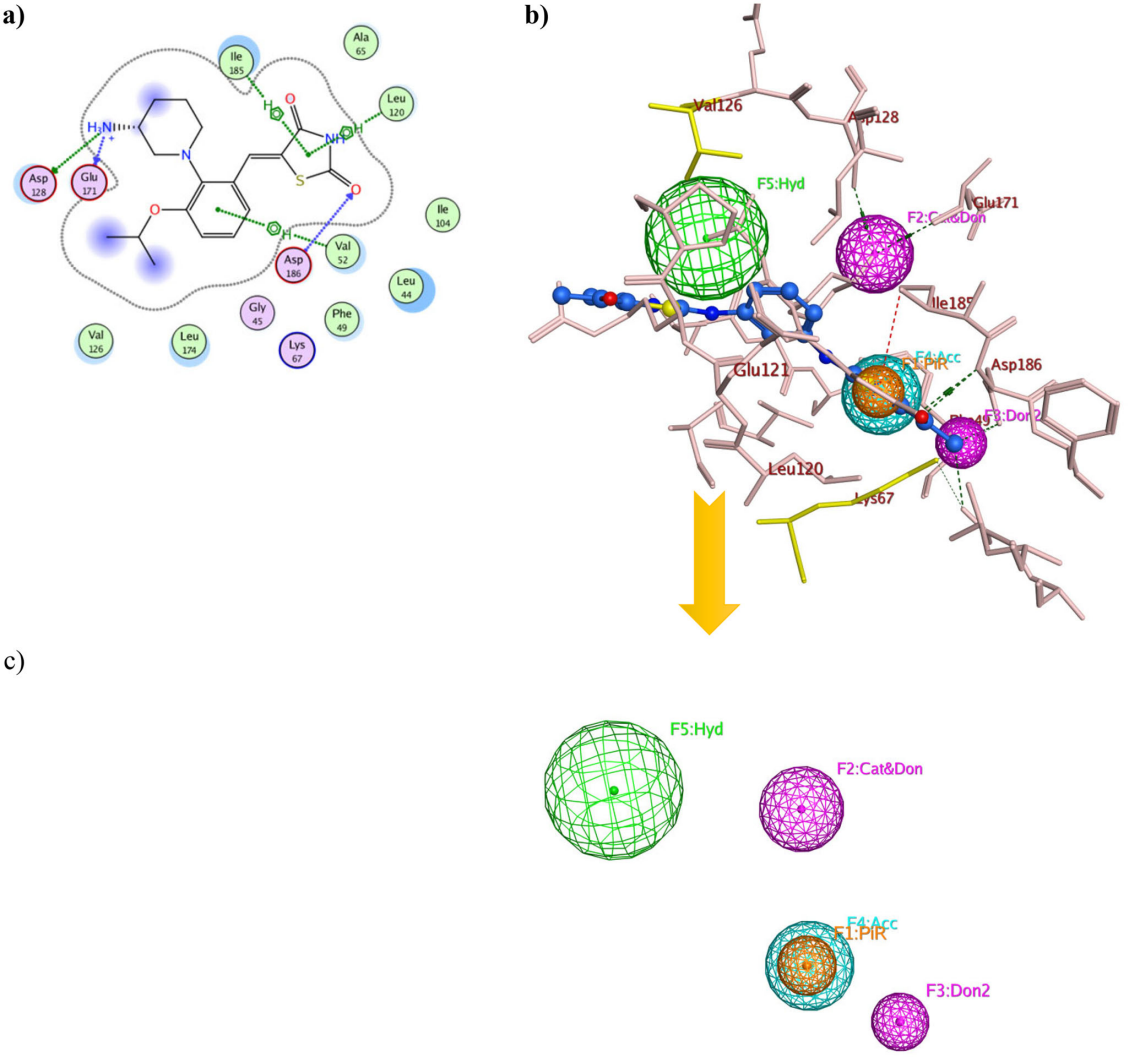
Three-dimensional presentation of the structure based pharmacophore model using PDB: 4DTK where (a) showed the 2D interaction of **7LI** inside the active site (b) showed **7LI**-Pim1 interaction pattern with its pharmacophore features while (c) was the calculated model.

Structure-based pharmacophore modelling was used to point out the essential electronic and steric features for the interaction with Pim1 binding site with the possible added features to interact with the catalytic Lys67 and the unique Val126 (Figure 6(a–c)). The Pim1 protein structure co-crystallised with thiazolidinedione derivative **7LI** (PDB 4DTK, resolution 1.86 Å) was downloaded to study its 2D interaction with the active site residues (Figure 6(a)). This interaction pattern was used to study the pharmacophore annotations which yielded a model of five features. Two of those features dictated the 7LI interaction with the binding site; the hydrophobic Л system and cationic/donor moieties represented as F1:PiR and F2:Cat&Don, respectively (Figure 6(b)) that bound to Ile185, Asp128 and Glu171, respectively. However; the design of potent and preferential Pim1 derivatives necessitated the addition of three other moieties to cope with the required interaction with Lys67 and Val126. As illustrated in Figure 6(b), the hydrogen bond forming and the hydrophobic features represented as F3:Don2, F4:Acc and F5: Hyd were critically added to the newly designed derivatives to interact with Lys67 and Val126, respectively (Figure 6(c)). This model was validated through a test set of 30 active and 10 inactive reported Pim1 inhibitors downloaded from the Binding Database^28^. The proposed derivatives complied with the constructed model showing RMSD range of 0.13–0.81.

## Experimental methods

### Chemistry

Melting points were carried out by the open capillary tube method using digital electrically heated GallenKamp^®^, England and uncorrected. Elemental microanalyses were carried out at the Regional Center for Mycology and Biotechnology, Al-Azhar University. Infra-red spectra were done on Schimadzu IR spectrometer 8400S and expressed in wave number (*υ*_max_) cm^−1^. The ^1^H NMR spectra were measured in dimethylsulphoxide (DMSO-*d*_6_) on Bruker AVANCE III 500 MHz FT-NMR spectrophotometer at King Khaled University, KSA and Drug discovery and research centre at Ain-Shams and Zagazig Universities using Bruker 400 MHz NMR spectrophotometer. Chemical shifts were recorded in δ as parts per million (ppm) shifted from tetramethylsilane (TMS) as internal standard. ^13^C NMR spectra were recorded at 125 MHz and 100 MHz. Mass spectra were performed on Hewlette Packard (HP)-5988 mass spectrometer at 70 eV at the Regional Center for Mycology and Biotechnology, Al-Azhar University. Reactions were monitored by thin layer chromatography (TLC) using Macherey-Nagel Alugram Sil G/UV 254 silica gel plates and ethylacetate: petroleum ether (2.5:2.5) as an eluting system unless otherwise stated. The spots were visualised using Vilber Lourmet lamp at *λ* = 254 and 365 nm.

Compounds **2**^29^^,^^30^ and **7**^31^ were prepared according to their reported procedures.

#### General synthesis procedures for 3a–c

To a stirred solution of 1,1′-(1,3-phenylene)*bis*(thiourea) **2** (2 mmol, 1 equiv.) in absolute ethanol (10 ml), 1-chloropropan-2-one derivative (4 mmol, 2 equiv.) was added with 3 drops of triethylamine. The reaction mixture was heated under reflux for 8 h. Upon reaction completion, the mixture was poured on ice/water then the precipitate was picked up and recrystallized from ethanol.

##### N^1^,N^3^-bis(4-methylthiazol-2-yl)benzene-1,3-diamine (3a)

Yellow powder (80%), mp. 137–139 °C; IR (cm^−1^, Nujol): 3200 (NH), 3005 (CH aromatic, stretching), 2954 (CH aliphatic), 1563 (C=C aromatic), 686 (CH aromatic, bending).^1^H NMR (DMSO-*d_6_*, 500 Hz, *δ*, ppm): 2.24 (6H, s, 2CH_3_), 6.44 (2H, s, thiazole -H5), 7.17–7.18 (3H, m, Ar-H), 7.93 (1H, s, phenyl –N–C=CH-C–N), 10.05 (2H, s, 2NH). ^13^C NMR (DMSO-*d_6_*, 100 MHz, *δ*, ppm):17.22 (aliphatic -CH_3_), 102.1, 104.9, 109.9, 129.2 (phenyl carbons), 141.8, 147.8, 162.9 (thiazole carbons). MS (*m*/*z*, %): 302.1 (100.0%). Found: 302.3 (8%), 77.1 (100%). Anal. Calcd. For C_14_H_14_N_4_S_2_: C, 55.60; H, 4.67; N, 18.53; S, 21.21; Found: C, 55.81; H, 4.80; N, 18.79; S, 21.08.

##### 1,1’-(2,2’-(1,3-Phenylenebis(azanediyl))bis(4-methylthiazole-5,2-diyl))diethanone (3b)

Brown powder (75%); mp 250–251 °C; IR (cm^−1^, Nujol): 3200 (NH), 2998 (CH aromatic, stretching), 2961 (CH aliphatic, stretching), 1655 (C=O), 1597 (C=C aromatic), 1352 (CH aliphatic, bending), 713 (CH aromatic, bending). ^1^H NMR (DMSO-*d_6_*, 400 Hz, *δ*, ppm): 2.25 (6H, s, aliphatic -CH_3_), 2.49 (6H, s, aliphatic COCH_3_), 7.19–7.33 (3H, m, phenyl-N-C=CH-CH=CH-C-N-), 8.05 (1H, s, phenyl -N-C=CH-C-N), 10.84 (2H, s, -NH, D_2_O exchangeable). ^13^C NMR (DMSO-*d_6_*, 100 MHz, *δ*, ppm):18.4 (aliphatic -CH_3_), 29.7 (aliphatic -COCH_3_), 106.9, 112.5, 122.5, 129.7 (phenyl carbons), 140.6, 156.2, 164.8 (thiazole carbons), 189.1 (-C=O). MS (*m*/*z*, %): 386.1 (100.0%). Found: 386.1 (25%), 127.1 (95%). Anal. Calcd. For C_18_H_18_N_4_O_2_S_2_: C, 55.94; H, 4.69; N, 14.50; O, 8.28; S, 16.59 Found: C, 56.15; H, 4.81; N, 14.68; S, 16.48.

##### Diethyl 2,2’-(1,3-phenylenebis(azanediyl))bis(4-methylthiazole-5-carboxylate) (3c)

Brown powder (60%); mp 107–108 °C; IR (cm^−1^, Nujol): 3200 (NH), 2998 (CH aromatic, stretching), 2961 (CH aliphatic, stretching), 1655 (C=O), 1597 (C=C aromatic), 1352 (CH aliphatic, bending), 713 (CH aromatic, bending).^1^H NMR (DMSO-*d_6_*, 400 Hz, *δ*, ppm): 1.32 (6H, t, *J* = 4.0 Hz, aliphatic -CH_2_CH_3_), 2.10 (6H, s, aliphatic -CH_3_), 4.22 (4H, m, *J* = 4.0 Hz, aliphatic -CH_2_CH_3_), 7.13–7.32 (3H, m, phenyl-N-C=CH-CH=CH-C-N-), 8.13 (1H, s, phenyl -N-C=CH-C-N), 10.79 (2H, s, -NH, D_2_O exchangeable). ^13^C NMR (DMSO-*d_6_*, 100 MHz, *δ*, ppm):14.3 (aliphatic -CH_2_CH_3_), 17.3 (aliphatic -CH_3_), 60.2 (aliphatic -CH_2_CH_3_), 112.2, 129.7 (phenyl carbons), 140.6, 161.8 (thiazole carbons), 175.0 (-C=O). MS (m/z, %): 446.1 (100.0%), 447.1 (25.1%). Found: 445.3 (96%), 446.3 (22%), 447.4 (17%).

#### General synthesis procedures for 5a–b

To a stirred solution of 1,1′-(1,3-phenylene)*bis*(thiourea) **2** (2 mmol, 1 equiv.) in absolute ethanol (10 ml), 2-oxo-*N*’-phenylpropanehydrazonoyl chloride derivatives **4a–c** (4 mmol, 2 equiv.) was added with 3 drops of triethylamine. The reaction mixture was heated under reflux for 8 h. Upon reaction completion, the mixture was poured on ice/water to yield crude products.

##### N^1^,N^3^-bis(4-methyl-5-(phenyldiazenyl)thiazol-2-yl)benzene-1,3-diamine (5a)

The crude product was purified by flash chromatography using normal phase and ethyl acetate: petroleum ether (5:5) as elution system to yield yellow powder (45%); mp 115–116 °C; IR (cm^−1^, Nujol): 3200 (NH), 3005 (CH aromatic, stretching), 1536 (C=C aromatic), 1490 (N = N), 825 (CH aromatic, bending). ^1^H NMR (DMSO-*d_6_*, 400 Hz, *δ*, ppm): 2.43 (6H, s, aliphatic -CH_3_), 7.11–7.73 (14H, m, aromatic -CH), 9.73 (2H, s, -NH, D_2_O exchangeable). ^13^C NMR (DMSO-*d_6_*, 100 MHz, *δ*, ppm): 38.8 (aliphatic -CH_3_), 128.4, 128.4, 128.5, 128.8, 128.9, 129.2, 130.0, 130.2, 130.6, 130.9, 131.2 (aromatic carbons). MS (*m*/*z*, %): 510.1 (100.0%) Found: 510.2 (45%), 77.4 (100%). Anal. Calcd. For C_26_H_22_N_8_S_2_: C, 61.15; H, 4.34; N, 21.94; S, 12.56 Found: C, 60.97; H, 4.50; N, 22.13; S, 12.70.

##### N^1^,N^3^-bis(5-((4-ethoxyphenyl)diazinyl)-4-methylthiazol-2-yl)benzene-1,3-diamine (5b)

The crude product was recrystallized from ethanol to yield yellowish brown powder (67%); mp 116–118 °C; IR (cm^−1^, Nujol): 3200 (NH), 3000 (CH aromatic, stretching), 2985 (CH aliphatic, stretching), 1610 (C=C aromatic), 1516 (N = N), 1324 (CH aliphatic, bending), 1245 (C-O ether), 795 (CH aromatic, bending).^1^H NMR (DMSO-*d_6_*, 400 Hz, *δ*, ppm): 1.38 (6H, t, aliphatic -CH_2_CH_3_), 2.45 (6H, s, aliphatic -CH_3_), 4.08–4.10 (4H, m, *J* = 8.0 Hz, aliphatic -CH_2_CH_3_), 7.06–7.08 (7H, m, *J* = 8.0 Hz, phenyl -CH), 7.56–7.58 (4H, m, *J* = 8.0 Hz, azo-phenyl -CH), 8.26 (2H, s, -NH, D_2_O exchangeable). ^13^C NMR (DMSO-*d_6_*, 100 MHz, *δ*, ppm): 26.4 (aliphatic -CH_2_CH_3_), 31.0 (aliphatic -CH_3_), 63.4 (aliphatic -CH_2_CH_3_), 108.8, 115.3, 117.9, 119.0, 129.0, 132.5, 135.0, 139.2, 156.7, 180.9 (aromatic carbons). MS (*m*/*z*, %): 598.2 (100.0%). Found: 2598.2 (10%), 77.3 (100%). Anal. Calcd. For C_30_H_30_N_8_O_2_S_2_: C, 60.18; H, 5.05; N, 18.71; O, 5.34; S, 10.71; Found: C, 59.91; H, 5.17; N, 18.94; S, 10.89.

#### General synthesis procedures for 6a–c

To a stirred solution of 1,1′-(1,3-phenylene)*bis*(thiourea) **2** (2 mmol, 1 equiv.) in absolute ethanol (10 ml), 2-chloro-1-phenylethanone derivative (4 mmol, 2 equiv.) was added with 3 drops triethylamine. The reaction mixture was heated under reflux for 8 h. Upon reaction completion, the mixture was poured on ice/water then the precipitate was recrystallized from ethanol.

##### N^1^,N^3^-bis(4-phenylthiazol-2-yl)benzene-1,3-diamine (6a)

White powder (60%); mp 92–94 °C. IR (cm^−1^, Nujol): 3200 (NH), 3023 (CH aromatic, stretching), 1607 (C=C aromatic), 703 (CH aromatic, bending). ^1^H NMR (DMSO-*d_6_*, 400 Hz, *δ*, ppm): 7.29–7.42 (10H, m, central phenyl-CH, *meta* and *para* terminal phenyl -CH), 7.94 (4H, d, *J* = 8.0 Hz, *ortho*-terminal phenyl -CH), 8.10 (2H, s, thiazole -CH), 10.31 (2H, s, -NH, D_2_O exchangeable). ^13^C NMR (DMSO-*d_6_*, 100 MHz, *δ*, ppm): 128.4, 128.5, 128.8, 128.9, 129.2, 130.1, 130.2, 130.6, 130.9, 131.2 (aromatic carbons). MS (*m*/*z*, %): 426.1 (100.0%), 427.1 (29.2%). Found: 425.2 (97.2%), 427.3 (20%).

##### N^1^,N^3^-bis(4–(4-fluorophenyl)thiazol-2-yl)benzene-1,3-diamine (6b)

Off-white powder (66%); mp 80–83 °C. IR (cm^−1^, Nujol): 3200 (NH), 3023 (CH aromatic, stretching), 1607 (C=C aromatic), 703 (CH aromatic, bending). ^1^H NMR (DMSO-*d_6_*, 400 Hz, *δ*, ppm): 7.11–7.29 (4H, m, *J* = 12.0 Hz, central phenyl-CH), 7.32–7.52 (6H, m, *J* = 12.0 Hz, *ortho*-phenyl -CH and thiazole -CH), 7.79–8.44 (4H, m, *J* = 12.0 Hz, *meta*-phenyl -CH), 10.32 (2H, s, -NH, D_2_O exchangeable). ^13^C NMR (DMSO-*d_6_*, 100 MHz, *δ*, ppm): 102.6, 105.5, 110.4, 115.4, 127.7, 129.5, 131.1, 141.8, 149.1, 160.6, 163.3 (aromatic carbons). MS (*m*/*z*, %): 462.1 (100.0%). Found: 462.8 (15%), 44.1 (100%) Anal. Calcd. For C_24_H_16_F_2_N_4_S_2_; C, 62.32; H, 3.49; F, 8.21; N, 12.11; S, 13.86; Found: C, 62.60; H, 3.71; N, 12.38; S, 13.95.

##### N^1^,N^3^-bis(4–(4-chlorophenyl)thiazol-2-yl)benzene-1,3-diamine (6c)

White powder (63%); mp 100–101 °C; IR (cm^−1^, Nujol): 3200 (NH), 3023 (CH aromatic, stretching), 1607 (C=C aromatic), 703 (CH aromatic, bending).^1^H NMR (DMSO-*d_6_*, 400 Hz, *δ*, ppm): 7.29 (4H, m, central phenyl-CH), 7.39–7.43 (4H, m, *J* = 12.0 Hz, *ortho*-phenyl -CH), 7.93–7.96 (4H, m, *J* = 12.0 Hz, *meta*-phenyl -CH), 8.17 (2H, s, and thiazole -CH), 10.33 (2H, s, -NH, D_2_O exchangeable). ^13^C NMR (DMSO-*d_6_*, 100 MHz, *δ*, ppm): 102.6, 105.5, 110.4, 115.4, 127.7, 129.5, 131.1, 141.8, 149.1, 160.6, 163.3 (aromatic carbons). MS (*m*/*z*, %): 494.0 (100.0%), 496.0 (77.1%), 495.0 (29.2%), 497.0 (21.6%). Found: 497.3 (24%), 495.3 (72%), 493.2 (94.8%).

#### General synthesis procedures for 8a–c

To a stirred solution of 1,1′-(1,3-phenylene)*bis*(3-phenylthiourea) **7** (2 mmol, 1 equiv.) in absolute ethanol (10 ml), 2-chloro-1-phenylethanone derivative (4 mmol, 2 equiv.) was added with 3 drops triethylamine. The reaction mixture was heated under reflux for 24 h. After cooling to RT, the obtained precipitate was filtered and the filtrate was poured on ice/water which crystallised and washed by ethanol.

##### N,N’-(3,3’-(1,3-phenylene)bis(4-phenylthiazole-3(3H)-yl-2(3H)-ylidene))dianiline (8a)

White powder (40%); mp= 110–111 °C; IR (cm^−1^, Nujol): 3100 (CH aromatic, stretching), 1600 (C=N), 1561 (C=C aromatic), 693 (CH aromatic, bending).^1^H NMR (DMSO-*d_6_*, 500 Hz, *δ*, ppm): 6.50–7.40 (26 H, m, aromatic -CH). ^13^C NMR (DMSO-*d_6_*, 100 MHz, *δ*, ppm): 128.2–130.0 (aromatic carbons). MS (*m*/*z*, %): 578.2 (100.0%). Found: 578.1 (30%), 77.1 (100%). Anal. Calcd. For C_36_H_26_N_4_S_2_; C, 74.71; H, 4.53; N, 9.68; S, 11.08; Found: C74.89; H, 4.61; N, 9.85; S, 11.20.

##### N,N’-(3,3’-(1,3-phenylene)bis(4–(4-fluorophenyl)thiazole-3(3H)-yl-2(3H)-ylidene))dianiline (8b)

White powder (55%); mp = 115–117 °C; IR (cm^−1^, Nujol): 3100 (CH aromatic, stretching), 1610 (C=N), 1561 (C=C aromatic), 693 (CH aromatic, bending).^1^H NMR (DMSO-*d_6_*, 400 Hz, *δ*, ppm): 6.73 (2H, s, thiazole), 7.14–7.52 (22H, m, aromatic -CH). ^13^C NMR (DMSO-*d_6_*, 100 MHz, *δ*, ppm): 128.4–131.2 (aromatic carbons). MS (*m*/*z*, %): 614.1 (100.0%). Found: 614.03 (30%), 77.2 (100%). Anal. Calcd. For C_36_H_24_F_2_N_4_S_2_; C, 70.34; H, 3.94; F, 6.18; N, 9.11; S, 10.43; Found: C, 70.59; H, 3.71; N, 9.32; S, 10.64.

##### N,N’-(3,3’-(1,3-phenylene)bis(4–(4-chlorophenyl)thiazole-3(3H)-yl-2(3H)-ylidene))dianiline (8c)

White powder (50%); mp= 120–121 °C; IR (cm^−1^, Nujol): 3100 (CH aromatic, stretching), 1610 (C=N), 1561 (C=C aromatic), 693 (CH aromatic, bending).^1^H NMR (DMSO-*d_6_*, 400 Hz, *δ*, ppm): 6.73 (2H, s, thiazole), 7.14–7.52 (22H, m, aromatic -CH). ^13^C NMR (DMSO-*d_6_*, 100 MHz, *δ*, ppm): 128.4–131.2 (aromatic carbons). MS (*m*/*z*, %): 646.1 (100.0%). Found: 644.9 (21.4%), 647.1 (M + 2, 9.5%). Anal. Calcd. For C_36_H_24_Cl_2_N_4_S_2_; C, 66.76; H, 3.74; Cl, 10.95; N, 8.65; S, 9.90; Found: C, 66.94; H, 3.85; N, 8.77; S, 9.78.

#### 1,1’-(3,3’-(1,3-Phenylene)bis(4-ethoxy-2-(phenylimino)-2,3-dihydrothiazole-5,3-diyl)diethanone (9)

To a stirred solution of 1,1′-(1,3-phenylene)*bis*(3-phenylthiourea) **7** (2 mmol, 1 equiv.) in absolute ethanol (10 ml), ethyl 2-chloro-3-oxobutanoate (4 mmol, 2 equiv.) was added with 3 drops triethylamine. The reaction mixture was heated under reflux for 24 h. Upon reaction completion, the mixture was poured on ice/water then the precipitate was recrystallized from ethanol to yield **9** as white powder (44%); mp = 116–118 °C; IR (cm^−1^, Nujol): 3000 (CH aromatic, stretching), 2900 (CH aliphatic), 1699 (C=O), 1620 (C=N), 1566 (C=C aromatic), 755 (CH aromatic, bending).^1^H NMR (DMSO-*d_6_*, 400 Hz, *δ*, ppm): 1.20 (6H, s, aliphatic -CH_3_), 2.40 (6H, s, CO-CH_3_), 6.88–7.60 (14H, m, phenyl -CH). ^13^C NMR (DMSO-*d_6_*, 100 MHz, *δ*, ppm):14.1 (aliphatic -CH_3_), 60.5 (aliphatic -COCH_3_), 120.6, 123.4, 129.0, 130.6, 136.3, 137.4, 147.2, 150.5, 151.6, 158.1, 161.0, 161.2 (-C=O). MS (*m*/*z*, %): 538.1 (100.0%). Found: 537.2 (15%), 227.8 (100%). Anal. Calcd. For C_30_H_26_N_4_O_2_S_2_: C, 66.89; H, 4.86; N, 10.40; O, 5.94; S, 11.90, Found: C, 67.12; H, 4.97; N, 10.63; S, 11.97.

### Pharmacophore modelling

The Molecular Operating Environment MOE^®^ 2014.0901were used as the *in silico* operating interface with amber10: EHT as the selected forcefield and EHT scheme that tethered at 5000. Pim1 protein X-ray crystal was downloaded from the protein data bank with PDB code 4DTK. The best quality chain was selected and all unnecessary moieties, solvent and ligands were removed. The protein structure was corrected and protonated at the default pH before proceeding to annotate the essential pharmacophore features based on the co-crystallised ligand.

### Cancerous cell line one-dose screening

The 60 cancerous cell panel screening was performed following the Developmental Therapeutics Program (DTP) of the National Cancer Institute (NIH-NCI), USA. A single dose of 10 µM was used to perform the screening at 48 h incubation and the results were achieved as cancerous cell growth percentage relative to the no-drug control that was converted to an inhibition percentage by subtracting from 100.The cancerous cell lines were grown in RPMI 1640 medium containing 5% foetal bovine serum and 2 mM l-glutamine at 37 °C, 5% CO_2_, 95% air and 100% relative humidity for 24 h prior to addition of the tested compounds. After 24 h, two plates of each cell line were fixed *in situ* with TCA to represent a measurement of the cell population for each cell line at the time of drug addition (*T*_z_). Compounds were dissolved in DMSO at 400-fold the desired final maximum test concentration and stored frozen prior to use. At the time of drug addition, an aliquot of frozen concentrate was thawed and diluted to twice the desired final maximum test concentration with complete medium containing 50 µg/mL gentamicin. Aliquots of 100 µL of the compounds dilution was added to the appropriate micro titre wells already containing 100 µL of medium, resulting in the required final drug concentrations of 10 µM. Following compounds addition, the plates were incubated for an additional 48 h at 37 °C, 5% CO_2_, 95% air, and 100% relative humidity. For adherent cells, the assay was terminated by the addition of cold TCA. Cells were fixed *in situ* by the gentle addition of 50 µL of cold 50% (w/v) TCA (final concentration, 10% TCA) and incubated for 60 min at 4 °C. The supernatant was discarded, and the plates were washed five times with tap water and air dried. Sulphorhodamine B (SRB) solution (100 µL) at 0.4% (w/v) in 1% acetic acid is added to each well, and plates are incubated for 10 min at room temperature. After staining, unbound dye was removed by washing five times with 1% acetic acid and the plates were air dried. Bound stain was subsequently solubilised with 10 mM trizma base, and the absorbance was read on an automated plate reader at a wavelength of 515 nm. For suspension cells, the methodology was the same except that the assay was terminated by fixing settled cells at the bottom of the wells by gently adding 50 µL of 80% TCA (final concentration, 16% TCA). Using the seven absorbance measurements [time zero, (Tz), control growth, I, and test growth in the presence of drug at the five concentration levels (Ti)], the percentage growth was calculated as [32]:

[(Ti−Tz)/(C−Tz)]×100 for concentrations for which Ti>/=Tz

 [(Ti−Tz)/Tz]×100 for concentrations for which Ti<Tz.

### In vitro inhibition concentration of breast T47D cancer cell line

Cells were seeded and treated with a range of inhibitors concentration 39–10,000 nM after 24 h relative to DMSO. Plates were fixed 96 h after treatment with crystal violet then re-suspended with methanol and the absorption was measured using plate reader [33].

### In vitro Pim1and Pim2 kinase inhibition assay

ADP-Glo^TM^ kinase assay kit was used to evaluate Pim1 kinase inhibition purchased from Promega Corporation, Madison, WI. At each of the kit low-volume well, the tested compounds were dissolved in DMSO at each of the tested concentration range and 1 µL was withdrawn and mixed with 2 µL Pim1 kinase and 2 µL of substrate/ATP mix then incubated at room temperature for 60 min. 5 µL of ADP-Glo reagent was added and incubated for another 40 min at room temperature. Finally 10 µL of kinase detection reagents was added and incubated for 30 min and the luminescence was recorded. Pim1 kinase buffer was prepared by mixing 40 mM Tris at pH 7.5, 20 mM MgCl_2_, 0.1 mg/mL BSA and 50 µM DTT. Staurosporine and DMSO were used as positive and negative control, respectively following the same procedure^34^^,^^35^.

### Breast T47D cancer cell cycle flowcytometry

After treatment with test compounds for 48 h and paclitaxel (1 µM) for 48 h as positive control, cells (10^5^ cells) are collected by trypsinization and washed twice with ice-cold PBS (pH 7.4). Cells are re-suspended in two millilitres of 60% ice-cold ethanol and incubated at 4 °C for 1 h for fixation. Fixed cells are washed twice again with PBS (pH 7.4) and re-suspended in 1 ml of PBS containing 50 µg/mL RNAase A and 10 µg/mL propidium iodide (PI). After 20 min of incubation in dark at 37 °C, cells are analysed for DNA contents using flow cytometry analysis using FL2 (λ_ex/em_ 535/617 nm) signal detector (ACEA Novocyte™ flowcytometer, ACEA Biosciences Inc., San Diego, CA). For each sample, 12,000 events are acquired. Cell cycle distribution is calculated using ACEA NovoExpress™ software (ACEA Biosciences Inc., San Diego, CA)^36^.

### In silico molecular docking evaluation

The X-ray crystal of Pim1 and Pim2 kinase protein structures were downloaded from the Protein Data Bank (RCSB: PDB), corrected and 3D protonated at cut-off 15 Å using amber10:EHT forcefield of Molecular Operating Environment (MOE 2014.0901) software. The unnecessary chains, water molecules and ligands were removed. For Pim2 kinase, the high quality chain B was used. The binding site was selected using the corresponding co-crystallised ligand atoms at grid of 4.5 Å and the docking protocol was validated by re-docking the co-crystallised ligands till achieving the lowest RMSD with the lowest binding energy score. The molecular docking protocol used triangle matcher, London dG, GBVI/WSA algorithm as the placement, rescoring function 1 and 2, respectively for both enzymes. The tested compounds were constructed using Chemdraw Ultra 12.0 then transferred as smiles to MOE builder window, added their hydrogens and calculated the possible partial charges before energy minimisation at the same forcefield using RMSD gradient of 0.1.

### In vivo cytotoxicity study

The National Cancer Institute (Cairo, Egypt) provided the 48 male Swiss albino mice utilised in this study, which ranged in weight from 25 to 30 g. Prior to the experiment, mice were given a week to settle into their new environment. A constant 23 °C temperature, 60% humidity, and a 12/12 light/dark cycle were maintained in stainless steel cages. Each cage housed five mice with a plenty of nourishing food and refreshing water were served. This *in vivo* study was approved from the ethical committee of the Azhar University, Faculty of Medicine, Egypt.

#### Ehrlich solid carcinoma model

A female mouse with an 8-day-old ascetic tumour was obtained from the National Cancer Institute on the day of induction, and Ehrlich ascites carcinoma (EAC) cells were isolated from the ascetic fluid of the animal (day 0). Then, regular saline was used to dilute the ascetic fluid (1:10). Each mouse received a subcutaneous injection of 0.2 ml of ascetic fluid containing 2.5 × 10^6^ EAC cells in the right thigh of the hind limb to induce Ehrlich solid-phase cancer.

#### Calculation of the lethal dose 50 (LD_50_)

The LD_50_ of compounds **3b** and **8b** were determined using a fixed-dose approach^37^; 12 male Swiss albino mice were divided into four groups (three mice per group) to calculate the LD_50_. Compounds **3b** and **8b** were administered intra-peritoneal (i.p.) to mice at dosages of 5, 10, 15, and 20 mg/kg. By giving these concentrations once to the animals and monitoring the signs of poisoning and deaths within 24 h, the lethal dose was identified. The animals were monitored continuously for the first two hours and then every four hours after that. After 24 h, all of animal deaths and survival appeared. After dose of 20 mg/kg from the two-compound administration all mice were dead, after dose of 15 mg/kg from compounds all mice were dead. LD_50_ was estimated in **8b** by 10 mg/kg and in **3b** by 14 mg/kg. The dose used in the experiment was 1/10 from the LD_50_.

#### Experimental design for the in vivo study

On day 11 following ESC inoculation, tumour formation was observed as a solid mass expanding in the right thigh of each animal. The mice were then randomly divided into four groups (*n* = 6)

- Group (1) acted as the positive control group and mice received an i.p. DMSO (2 mg/kg/once weekly) for 3 weeks.
- Group (2) (Doxorubicin Group) mice received an i.p. dose of doxorubicin (2 mg/kg/once weekly) for 3 weeks^38^.
- Group (3) mice were given an i.p. dose of 8b compound (1 mg/kg/once weekly) for 3 weeks,
- Group (4) mice were given an i.p. dose of 3b compound (1.4 mg/kg/once weekly) for 3 weeks.

#### Histological examination

Two days after the last dose of therapy, blood was drawn from the mice’s orbital canthus, and serum was separated and stored at −80 °C for upcoming biochemical investigations. Following the cervical dislocation method of euthanasia, tumour samples from each mouse were collected and preserved in neutral buffered formalin at a 10% concentration for immunohistochemistry and histopathology studies. Histological preparations were performed according to the reported method^39^. Slices of Ehrlich tumour were cut to a thickness of 3–4 mm before being fixed in 10% neutral buffered formalin (10% NBF), dehydrated through a series of ethanol solutions, cleaned in xylene, and finally embedded in paraffin. To examine the overall tissue architecture, sections (4–6 m) thick were cut from paraffin blocks using a microtome and stained with haematoxylin and eosin. We used a Leica microscope (CH9435 Hee56rbrugg) to look at H&E-stained tissue sections (Leica Microsystems, Switzerland).

#### Immunohistochemistry staining protocol

The avidin–biotin–peroxidase complex (ABC) method was used to do immunohistochemistry on paraffin sections that were put on positively charged slides^40^. Rabbit Anti-VEGF Receptor 2 Polyclonal Antibody (Elabscience Cat# E-AB-63481, Dil.: 1:100) and Rabbit Monoclonal anti-caspase-3 antibody, active (cleaved) form (Sigma Aldrich Catalog # AB3623)^41^, were utilised during this IHC assessment. Each group’s sections were treated with the previously specified antibodies, after which the ABC technique reagents (Vectastain ABC-HRP kit, Vector labs) were applied. To locate the antigen–antibody combination, marker expression was stained with diaminobenzidine (DAB, manufactured by Sigma) and labelled with peroxidase. In order to conduct negative controls, non-immune serum was substituted for the primary or secondary antibodies. IHC-stained sections were assessed using a Leica (CH9435 Hee56sbrugg) microscope (Leica Microsystems, Switzerland).

### Statistical analysis

Using one-way analysis of variance (ANOVA) followed by Tukey’s post-hoc test, the difference between experimental groups was analysed. All data are presented as mean ± standard deviation (SD). All comparisons were judged statistically significant when the *p* values ≤ 0.05. ImageJ (NIH Image J system, Bethesda, MD) was used for the analysis of immunohistochemistry photos.

### Normal cell line cytotoxicity using MCF10a^42^

*In vitro* toxicology assay kit (MTT based) stock no. TOX-1 7H258 was purchased from SIGMA (St. Louis, MO). About 3 ml of the medium with the normal cell lines in log phase were added in multiwall plate without phenol red and serum. A volume of reconstituted MTT (#M-5655) equivalent to 10% of the culture medium volume was added then incubated for 2 h. The resulted fomazan crystals were dissolved with occasional shaking by equivalent volume of MTT solubilising solution (#M-8910). The final solution absorbance was measured spectrophotometry at wavelength 570 nm. Blank experiment was performed using medium only without cells.

## Results and discussion

### Chemistry

**Scheme 1.**
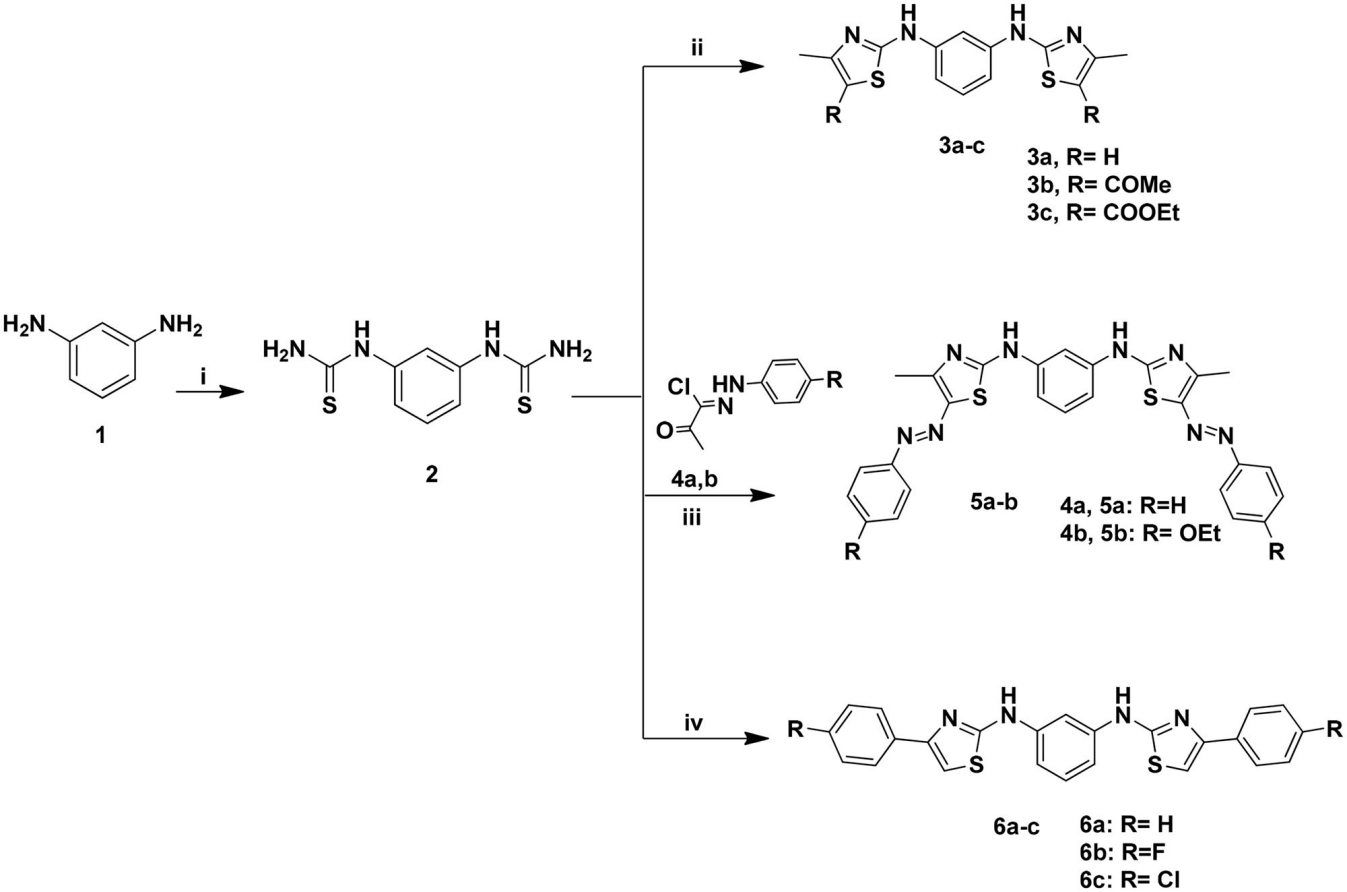
Synthesis of **3a–c**, **5a–b** and **6a–c**; Reagents and conditions: (i) NH_4_SCN (3eq.), H_2_O, HCl, reflux, 6 h; (ii) CH_3_COCHClR (2eq.), EtOH, TEA, reflux, 8–12 h; (iii) **4a–b** (2eq.), EtOH, TEA; (iv) 2-chloro-1-phenylethanone derivative (2eq.), EtOH, TEA, reflux, 24 h.

**Scheme 2.**
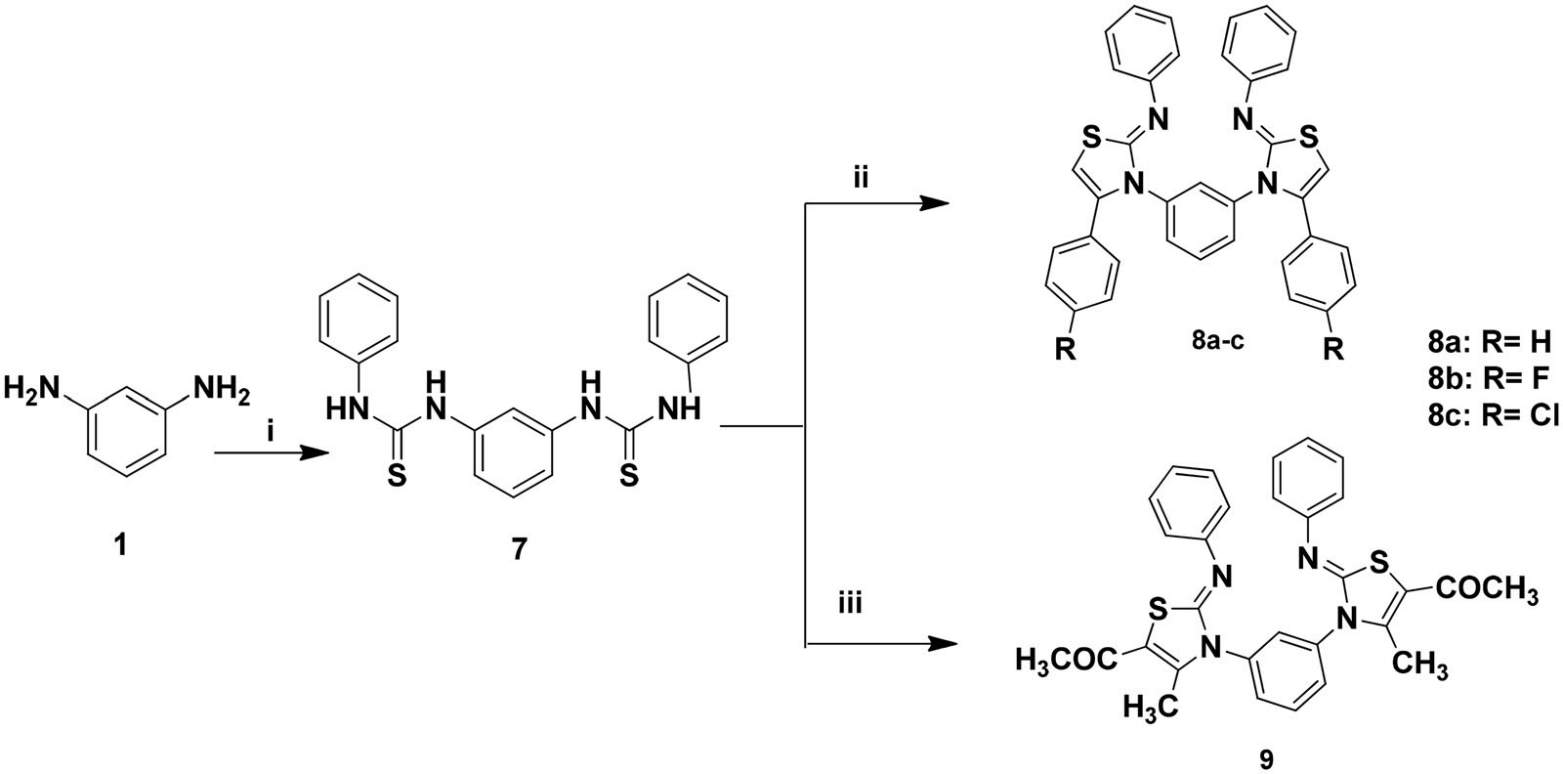
Synthesis of **8a–c** and **9**; Reagents and conditions: (i) PhNCS (2 eq.), EtOH, reflux, 2 h; (ii) 2-chloro-1-phenylethanone derivative (2 eq.), EtOH, TEA, reflux, 24 h; (iii) CH_3_COCHClCOCH_3_ (2eq.), EtOH, TEA, reflux, 24 h.

The targeted bis-thiazole derivatives **3a–c, 5a, 5b, 6a–c, 8a–c** and **9** were synthesised according to Schemes 1 and 2 where their precursors **2, 4a, 4b** and **7** were accessed following the reported methods^29–31^^,^^43^. Both schemes obeyed the Hantzsch condensation^44^^,^^45^ between different α-haloketones with the thioamides **2** and **7** which in turn were synthesised from the reaction of 1,3-diaminobenzene **1** with ammonium thiocyanate and phenyl isothiocyanate, respectively.

The achieved ^13^C NMR spectra of the synthesised derivatives had confirmed the presence of the thiazole carbons at *δ_c_* 140–165 ppm. Furthermore, the IR spectra confirmed the presence of the azo bond stretching band (-N = N-) of **5a** and **5b** at ύ 1490–1516 cm^−1^ and the imine stretching band of **8a–c** and **9** at ύ 1600–1620 cm^−1^.

It was noticed that the amine protons of **3a–c** and **6a–c** had appeared at *δ_H_* 10.0–10.84 ppm while for **5a** and **5b** were resonated at 8.26–9.73 ppm that were all D_2_O exchangeable. Moreover, analogues with di-substituted thiazole **3a** and **6a–c** showed their significant thiazole proton at *δ_H_* 6.44 and 7.3–8.1 ppm, respectively. Similarly, analogues **8a–c** exhibited their thiazole proton amongst the aromatic protons at *δ_H_* 7.14–7.52 ppm as appeared in the signals integration.

On the other hand, derivatives **3b** and **3c** were distinguished with their carbonyl substituents that showed prominent peaks in their IR and ^13^C NMR spectra at ύ 1655 cm^−1^ and *δ_c_* 188–189.1 ppm, respectively. In addition to their aliphatic protons which appeared at *δ_H_* 2.25–2.49 ppm and 1.32–4.22 ppm for **3b** and **3c**, respectively. Their corresponding aliphatic carbons were displayed at *δ_c_* 18.4–29.7 ppm and 14.3–60.2 ppm for **3b** and **3c**, respectively. Similar to **3b**, derivative **9** possessed a methyl ketone and a methyl substitution that appeared at *δ_H_* 1.2–2.4 ppm and *δ_c_* 14.1–60.5 ppm for methyl and methyl ketone, respectively. From their mass spectrum, compounds **5a**, **5b, 6a**, **6b, 8a** and **8b** showed the phenyl ring fragment at *m*/*z* 77. Moreover, the phenyl imine (Ph-N=) bond formation of **8a–c** was confirmed by the presence of their mass fragments at *m*/*z* 91–92. It was noticed that **5a** had poor solubility in DMSO that reflected weak signals in both NMR spectra.

### Cancerous cell line cytotoxicity

**Figure 7.**
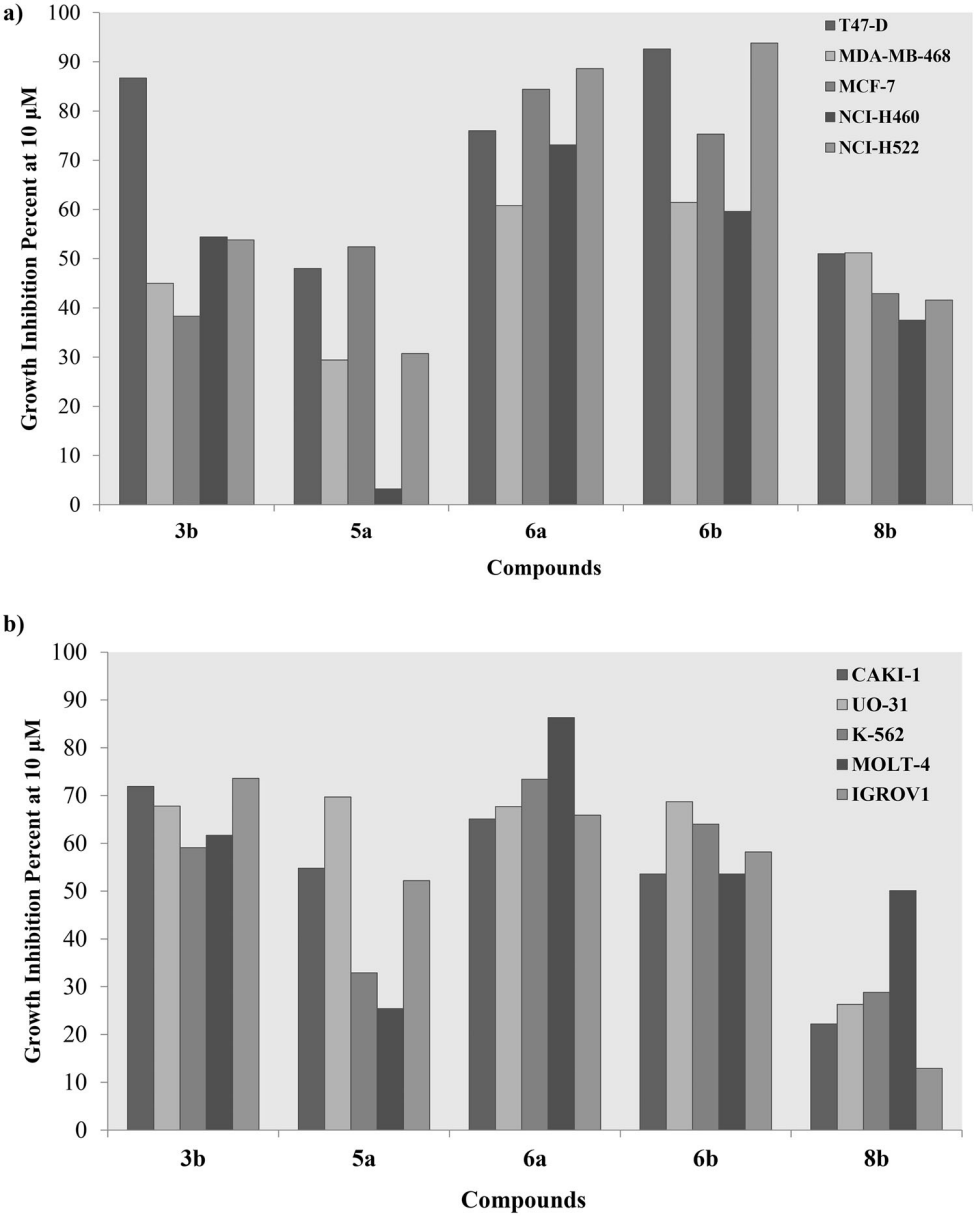
Growth inhibition percentage of **3b, 5a, 6a–b and 8b** using breast, non-small cell lung cancer cell lines (a), renal, leukaemia and ovarian cancer cell lines (b) at single concentration of 10 μM.

A preliminary screening of the synthesised compounds **2–9** cytotoxicity was done by the United State National Cancer Institute over a panel of 60 different cancerous cell lines at a single concentration of 10 µM. A summary of the most promising cytotoxic bis-thiazole derivatives with their responsive cancer cell was presented in Figure 7(a,b).

Amongst the tested breast cancer cell lines; the ductal carcinoma T47D was the most responsive towards treatment with 10 µM of derivatives **3b, 5a, 6a–b** and **8b** showing growth inhibition range of 48–92.6%. Nonetheless, the adenocarcinoma MCF-7 and breast cancer MDA-MB-231 showed an acceptable growth inhibition by **6a** and **6b**, respectively with 84.4% and 61.4%, respectively (Figure 7(a)). Similarly, derivatives **3b** and **6a–b** had a noticeable effect on the growth of two non-small cell lung cancer cell lines NCI-H460 and NCI-H522 with **6a–b** resulted in 88.6% and 93.8% inhibition of NCI-H522, respectively (Figure 7(a)). On the other hand, derivatives **3b** and **6a–b** exhibited a remarkable inhibition of the leukaemia K562 and MOLT-4 growth with the best inhibition was achieved by **6a** of 73.4% and 86.3%, respectively (Figure 7(b)). Likewise, **3b**, **5a** and **6a–b** revealed a growth inhibition range of 53.6–71.9% for the renal CAKI-1 cell line. Interestingly, the four derivatives almost had the same inhibition potential on the other renal UO-31 cell line with an approximate percentage of 68.7% (Figure 7(b)). Additionally, ovarian IGROV1 cell line growth was decreased upon treating with **3b** and **6a** by 73.6% and 65.9%, respectively.

Structurally, the aliphatic substituted phenyl aminothiazole derivatives **3a–c** showed remarkable decrease of their cytotoxic activity if unsubstituted or substituted with an ester group at **3a** and **3c**, respectively. Meanwhile, adding a methyl ketone at **3b** resulted in more than 50% growth inhibition of several cancerous cell lines at the tested concentration (Table S1). On the other hand, the un/substituted azophenyl derivatives **5a–b** showed lower cytotoxicity than their aliphatic congeners where the unsubstituted **5a** had noticeable cytotoxicity towards renal CAKI-1, SN12C and UO-31 cell lines comparing to its ether analogue **5b**. Removing the azo-linker and directly attach the phenyl moiety to the thiazole ring at **6a–c** favoured their cytotoxicity with the unsubstituted and *para*-fluoro substitution offered inhibitory activity of several cell line growth comparing to their chloride congener. In the same context; replacing the azo-linker with an imine at **8a–c** increased the cytotoxicity towards MCF-7 in the presence of another *para*-fluoro phenyl. Replacing the third phenyl group of the imine containing derivatives **8a–c** with aliphatic moieties **9** decreased its cytotoxic activity exhibited only 59% inhibition of the lung cancer HOP-92 cell line. Moreover, it was noticed that substituting the phenyl ring with a fluoride group had the privilege over the unsubstituted and chloride substituted analogues in **6a–c** and **8a–c**. This might due to the large size of the chloride moiety comparing to the hydrogen or fluoride that hindered the cellular uptake.

**Table 1.** Breast cancer T47D cell line IC_50_ values of **3a–b, 5a, 6a–c** and **8b** in μM at 48 h and 96 h incubation intervals and normal epithelia breast cell line IC_50_ of **3b** and **8b** in μM at 48 h using staurosporine as reference with the lowest values were highlighted in bold.

Compounds	Breast T47D cancer cell line		Breast MCF10A normal cell line	
	IC_50_ (μM, 48 h)	IC_50_ (μM, 96 h)	IC_50_ (μM, 48 h)	Selectivity Index
**3a**	7.82	2.86	**NT**	**NT**
**3b**	**5.77**	**1.46**	**105.33**	**18.25**
**5a**	10.40	4.35	**NT**	**NT**
**6a**	6.58	**1.78**	**NT**	**NT**
**6b**	5.92	**1.61**	**NT**	**NT**
**6c**	27.3	1.85	**NT**	**NT**
**8b**	**9.8**	**2.61**	**53.33**	**5.44**
**Staurosporine**	**4.34**	**NT**	**45.80**	**10.55**

As the most responsive cell line, breast T47D cancer cell line was used to further investigate the IC_50_ of derivatives **3a–b, 5a, 6a–c** and **8b** which displayed more than 50% growth inhibition at 10 µM. Their IC_50_ values were determined over 48 h and 96 h interval using the usual MTT assay (Table 1). The best achieved IC_50_ (96 h) were 1.46, 1.78, 1.61 and 2.61 µM by **3b, 6a–b** and **8b**, respectively. Therefore, those four derivatives were subsequently evaluated for their Pim1 kinase inhibition activity.

### In vitro Pim1 kinase inhibition

Based on the preliminary screening of the synthesised compounds′ ability to inhibit breast cancer T47D cancerous cell growth; derivatives **3b, 6a–b** and **8b** were picked for further evaluation of their Pim1 and Pim2 kinase inhibition using staurosporine as reference. The achieved *in vitro* kinases IC_50_ results were presented in Table 2. The naturally occurring staurosporine was selected as a broad spectrum serine/threonine kinase inhibitor to be the positive control^46^^,^^47^. Interestingly both **3b** and **8b** showed better enzymatic activity inhibition than staurosporine with IC_50_ 0.32, 0.24 and 0.36 µM, respectively. The high molecular weight of **8b** (614.1 g/mol) might have hindered its cellular uptake which was translated into a high cell line IC_50_ relative to a lower Pim1 IC_50_ upon using the *in vitro* enzyme assay kit (Tables 1 and 2). On the other hand, adding two phenyl groups to **3b** at each of its thiazole ring in **6a–b** caused dramatic increase in Pim1 IC_50_ despite their acceptable cell line inhibition. This could be explained by the lipophilicity increment of **6a–b** to improve the cancerous cell uptake however, not enough for proper positioning inside the active site of Pim1 and its consequent inhibition as detailed latter in the molecular docking section.

**Table 2.** Pim1 and Pim2 kinase IC_50_ values of **3b, 6a–b** and **8b** in μM with the lowest values were highlighted in bold.

Compounds	Pim1 kinase		Pim2 kinase	
	IC_50_ (μM, *n* = 3)	±SD	IC_50_ (μM, *n* = 3)	±SD
**3b**	**0.32**	0.02	6.05	0.32
**6a**	2.02	0.11	NT*	NT
**6b**	2.95	0.17	NT	NT
**8b**	**0.24**	0.01	10.53	0.02
**Staurosporine**	0.36	0.02	0.44	0.01

NT*: not tested; *n*: number of experiment replicates.

To investigate their selectivity towards Pim1, both **3b** and **8b** were further subjected to Pim2 inhibition evaluation (Table 2). Comparably, as predicted from their design, both derivatives showed higher Pim2 IC_50_ than their corresponding Pim1 IC_50_ giving 10.53 µM and 6.05 µM for **3b** and **8b**, respectively. The IC_50_ for Pim2 is higher 20 times than Pim2 for **3b** and 40 times for **8b** that rendered them promising hits for further optimisation to achieve more potent and preferential Pim1 inhibitor.

### Breast T47D cell cycle flowcytometry

**Figure 8.**
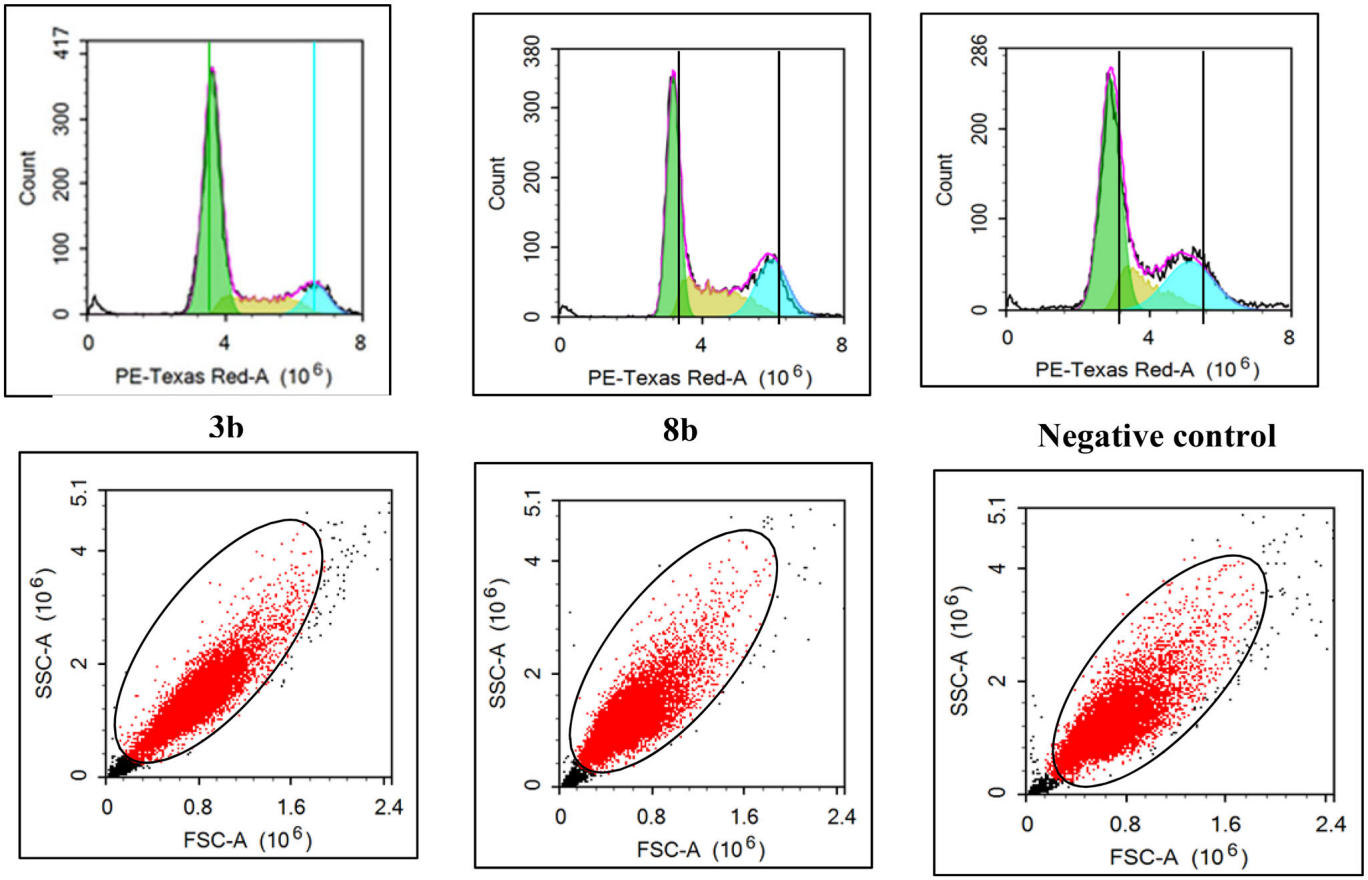
The effect of **3b** and **8b** on cell cycle distribution of the breast cancer T47D cell line after 48 h incubation.

**Figure 9.**
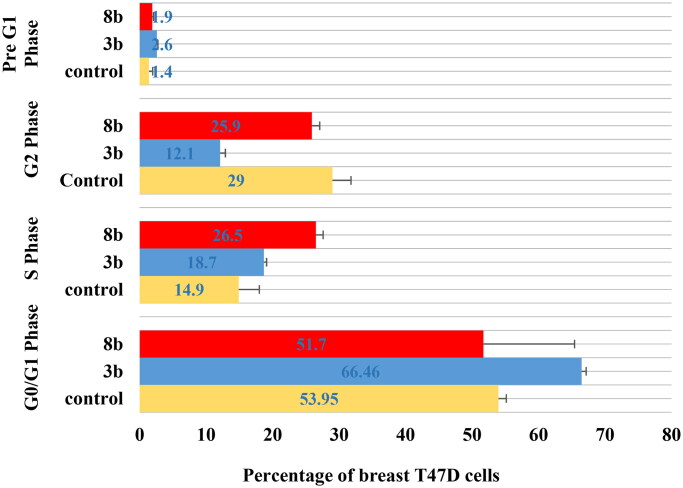
The percentage of cells that accumulate at each stage of breast cancer T47D cell line development was shown in a bar chart. All data are shown as the mean of three independent experiments ± standard deviation. One-way ANOVA test followed by Tukey’s post-hoc test at (*p* < 0.05) was used for statistical analysis.

The untreated control breast T47D cell lines showed rapid growth at G0/G1, S, G2 and sub G1 phase with 53.9%, 14.9%, 29.1% and 1.4%, respectively. The impact of the promising derivatives **3b** and **8b** on cell cycle distribution showed mainly at the early phases (Figures 8 and 9). At G0/G1 phase, **3b** and **8b** revealed 66.5% and 51.7% cells, respectively, which were slightly higher than the control group (53.9%).However, both **3b** and **8b** showed remarkable increment at S phase against the untreated cells with 18.7%, 26.5% and 14.9%, respectively, which was significantly higher than the control by 77.8% in case of **8b**. In the same context, during pre-G1 phase both **3b** and **8b** displayed 2.6% and 1.9%, respectively, with higher value than the untreated control group in case of **3b** for example by 78%. On the contrary, at G2 phase **3b** and **8b** presented only 12.2% and 25.9%, respectively, which were lower than the control cell lines for example by 58% in case of **3b**. Accordingly, **3b** proclaimed remarkable inhibition effect at the G0/G1-phase while **8b** affected mainly the S phase. These achieved results complied with the Pim1 kinase inhibition role at the cell cycle affirming the main effect of **3b** and **8b** at the early stages of the cell cycle.

### In silico molecular docking

**Figure 10.**
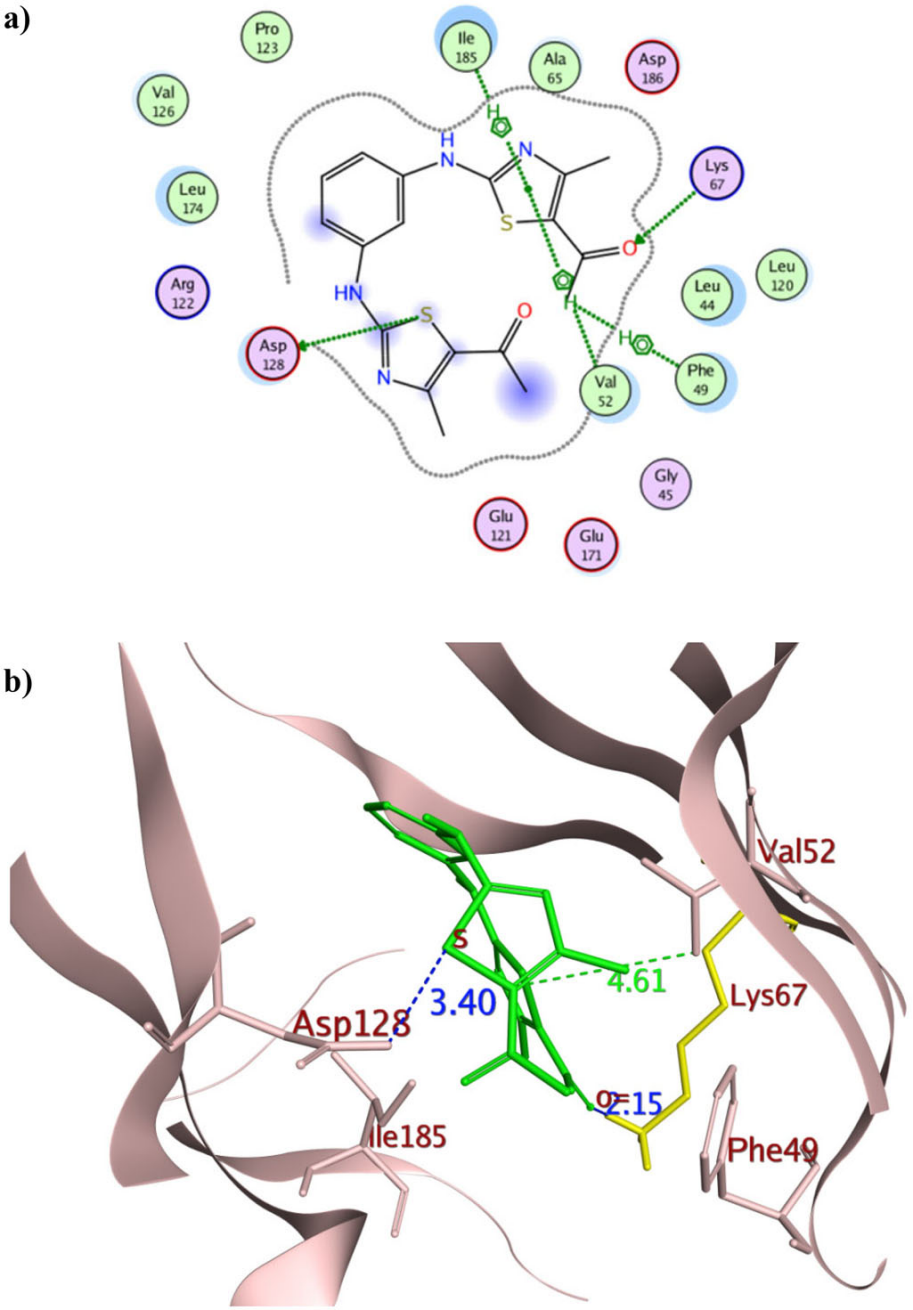
The 2D and 3D representations of molecular interactions of **3b** (a,b) as green stick model with Pim-1 kinase using PDB 4DTK where the conserved Lys67 at the active site and Val126 were highlighted as yellow stick model. The 3D representations highlighted the formed hydrogen bonds and H-pi interactions as blue and green dotted lines, respectively with their corresponding distance in Å.

**Figure 11.**
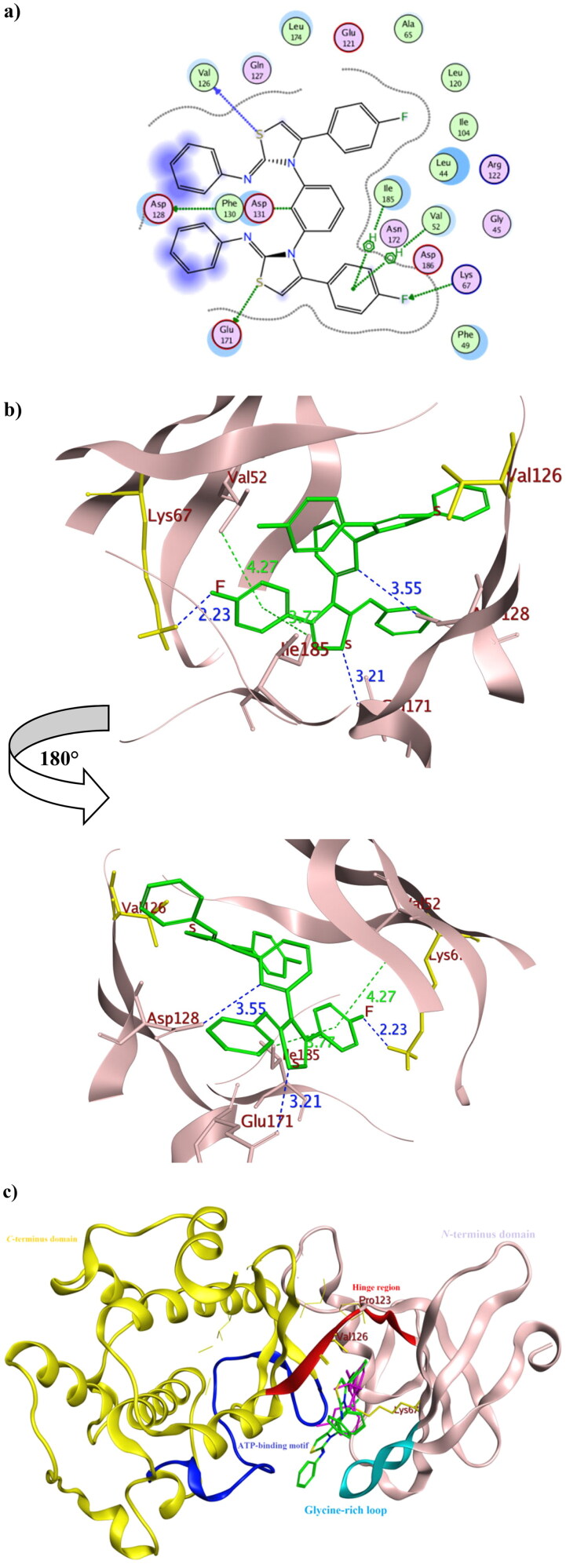
The 2D and 3D representations of molecular interactions of **8b** (a, b) as green stick model with Pim-1 kinase using PDB 4DTK where the conserved Lys67 at the active site and Val126 were highlighted as yellow stick model. The 3D representations highlighted the formed hydrogen bonds and H-pi interactions as blue and green dotted lines, respectively with their corresponding distance in Å. The position of the suggested orientation of **8b** relative to the co-crystallised ligand **7LI** was illustrated inside the full kinase protein structure in (c) mentioning the important kinase fragments.

To get further insights on the possible binding conformations of the active derivatives **3b** and **8b**; molecular docking was performed using the X-ray crystal of Pim1 kinase (PDB 4DTK) bound to the thiazolidinedione derivative **7LI**. The molecular docking protocol was validated before commencing the actual docking procedure by re-docking the co-crystallised **7LI** giving the lowest RMSD of 0.87. The achieved molecular docking results were summarised in Table 3 with the interaction patterns illustrated in Figures 10 and 11.

**Table 3.** The pharmacophore RMSD and molecular docking results of **3b** and **8b** using Pim1 kinase PDB: 4DTK with the crucial amino acids in bold.

Compound	Pharmacophore RMSD	Binding energy score (Kcal/mol)	Interacting atom of the ligand	Interacting amino acids	Interaction type	Bond distance (Å)
**7LI**	NA*	**−8.61**	N1	Asp128	H-donor	2.93
			N1	Glu171	H-donor	3.03
			O2	Asp186	H-acceptor	2.93
			N1	Asp128	Ionic	2.93
			6-ring	Val52	pi-H	4.74
			5-ring	Leu120	pi-H	3.93
			5-ring	Ile185	pi-H	4.13
**3b**	**0.32**	**−9.36**	S	Asp128	H-donor	3.40
			O	**Lys67**	**H-acceptor**	**2.15**
			C	Phe49	H-pi	3.72
			5-ring	Val52	pi-H	4.61
			5-ring	Ile185	pi-H	4.53
**8b**	**0.65**	**−10.39**	C	Asp128	H-donor	3.55
			S	**Val126**	**H-donor**	**4.15**
			S	Glu171	H-donor	3.21
			F	**Lys67**	**H-acceptor**	**2.23**
			6-ring	Val52	pi-H	4.27
			6-ring	Ile185	pi-H	3.77

NA*: not available.

The promising derivatives **3b** and **8b** showed better binding energy range to Pim1 active site than the co-crystallised ligand **7LI** giving −9.36, −10.36 and −8.61 Kcal/mol, respectively, with various types of interaction with the crucial residues (Table 3). The binding orientation of **3b** and **8b** inside Pim1 active site explained their astonishing *in vitro* IC_50_ giving 0.32 and 0.24 µM, respectively (Figures 10 and 11). Both **3b** and **8b** had the privilege over **7LI** in forming hydrogen bonds with the conserved active site Lys67 through their carbonyl oxygen and fluoride substitution, respectively. Their corresponding bond lengths were 2.15 and 2.23 Å for **3b** and **8b**, respectively, that considered stable enough to interfere with Lys67 catalytic activity^16^^,^^35^^,^^48^. It was of interest that **8b** interacted with the terminal amine group of the basic Lys67 through its fluorine substitution as H -bond acceptor albeit being rarely occurred^49^^,^^50^. Moreover, **8b** shared the interaction pattern of **7LI** with the active site residues Asp128 and Glu171 through H- bonding with the central phenyl and the sulphur atom of one of the thiazole rings, respectively, with an average length 3.0 Å (Figure 11(a, b)). Similarly, derivatives **3b** shared the interaction pattern of **7LI** with Ile185 through its thiazole ring hydrophobic interaction. Furthermore, **3b** showed additional **7LI**-like H- bond interactions with Asp128 through its sulphur atom of one of the thiazole rings.

Interestingly; it was assumed that interacting with the unique Val126 at the hinge region might offer a preferntial Pim1 inhibitor^17^ as it was replaced by Ala122 at Pim2 despite the 60% sequence similarity between both isoforms^18^. Consequently, **8b** had the potential to be a preferential inhibitor that needs further investigation and optimisation. Moreover, analogues **3b** showed better positioning at the active site through hydrophobic contact with Phe49 and Val52 at the glycine-rich loop. Phe49 served as a lid over the binding site thus its binding to **3b** hindered the access of ATP to its binding site and could hinder any possible conformational change of the apoprotein to accommodate its natural substrate by fixing Phe49 position^51^.

**Figure 12.**
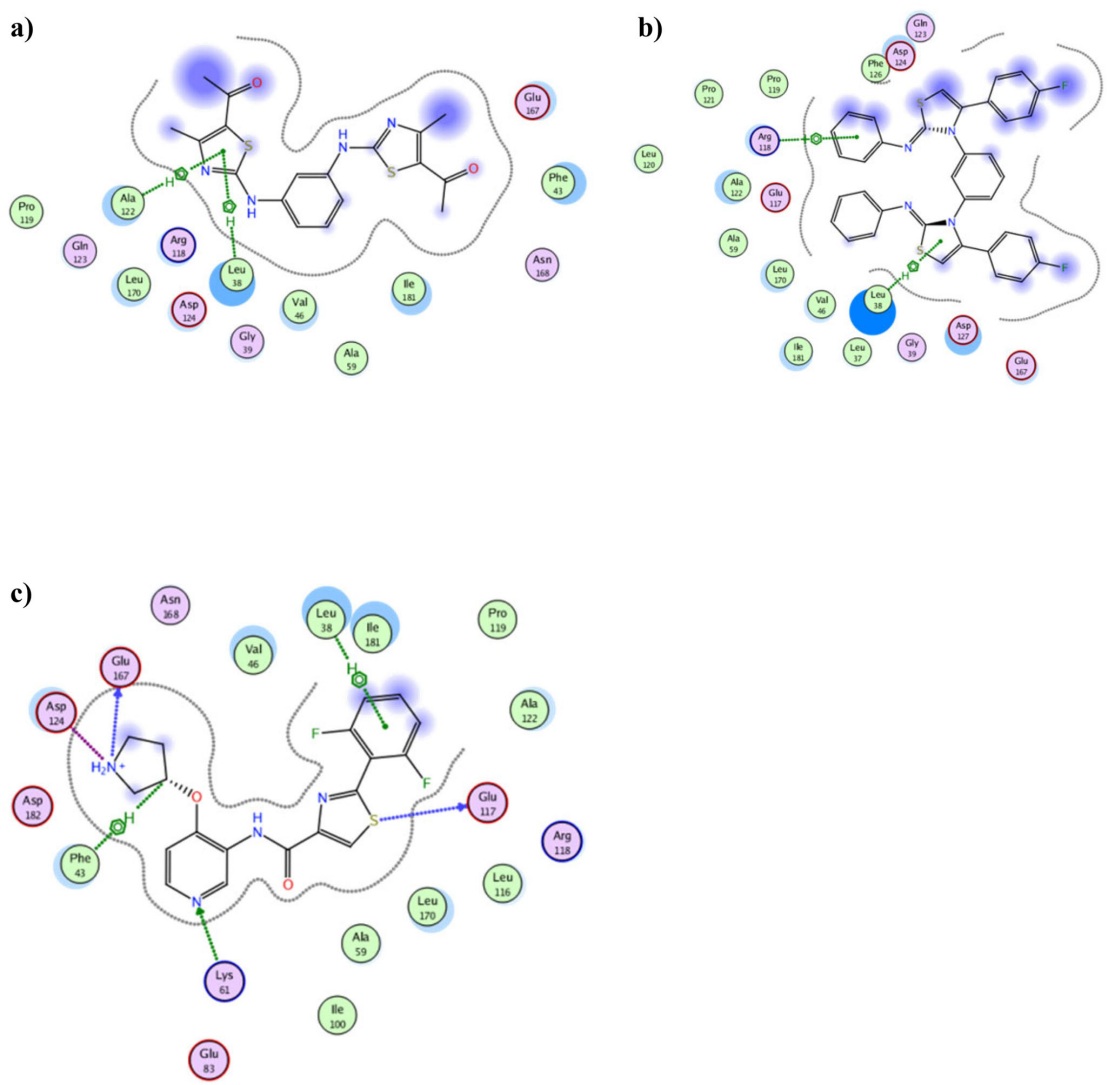
The 2D presentations of the molecular docking of **3b** (a), **8b** (b) and the co-crystallised ligand **3YR** (c) using Pim2 kinase PDB 4X7Q.

To *in silico* explain the selectivity of both **3b** and **8b** towards Pim1, molecular docking was performed using Pim2 PDB 4X7Q (resolution 2.33 Å)^52^ (Figure 12). The achieved docking results explained the *in vitro* IC_50_ of **3b** and **8b** which despite the formed hydrophobic interaction exhibited between **3b** and **8b** with Leu38, their distance exceeded 4.0 Å which classified as weak interaction. Moreover, the number and types of **3b** and **8b** interactions with Pim2 active site were considerably few when compared to the co-crystallised thiazole derivative **3YR** (Figure 12(a–c)). Additionally, **3b** and **8b** showed higher binding energy than **3YR** giving −8.50, −9.74 and −10.02 Kcal/mol, respectively, which together supported the suggestion of being preferably Pim1 inhibitor.

### In vivo cytotoxicity of 3b and 8b

#### Histopathological examination

**Figure 13.**
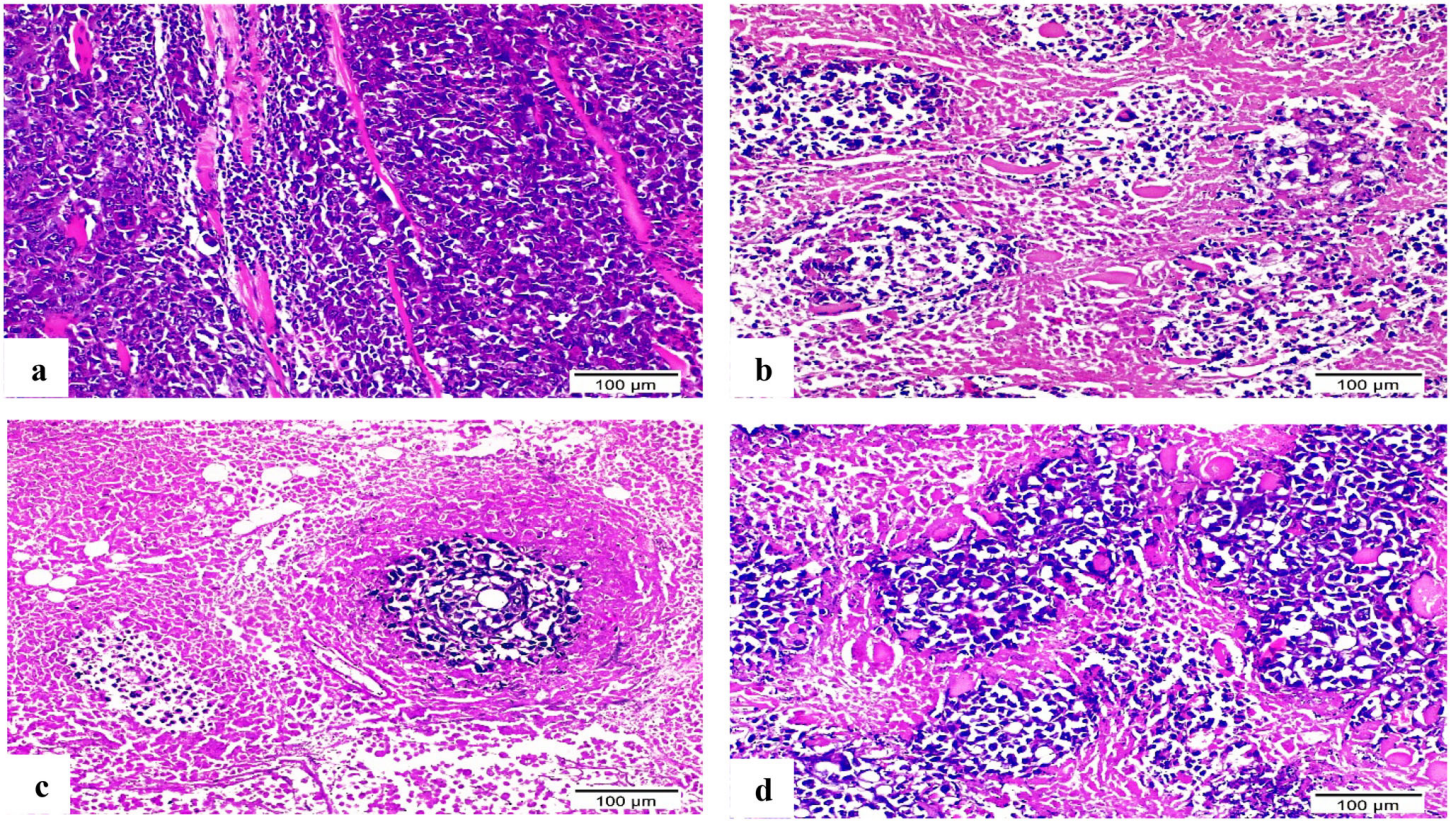
H&E-stained sections of Ehrlich solid tumour tissue (×200). (a) Untreated control demonstrated extensive tumour cell infiltration between individual muscle fibres. (b) Doxorubicin exposed small clusters of cancerous cells. (c) **8b** showed minimal infiltration of neoplastic cells and a significant reduction in their quantity. (d) **3b** displayed aggregates of neoplastic cells with their typical structure that surrounded by small patches of necrotic muscle tissue.

The Ehrlich tumour haematoxylin and eosin-stained sections highlighted the extensive tumour cell infiltration between individual muscle fibres (Figure 13(a)). The doxorubicin (DOX) treated group’s variant thigh section revealed a large region of necrotic muscle tissue surrounded by small clusters of cancerous cells (Figure 13(b)). Compound **8b** demonstrated muscle fibre with minimal infiltration of neoplastic cells and a significant reduction in their quantity (Figure 13(c)). In contrast, a substantial number of aggregated neoplastic cells with their typical structure were surrounded by small patches of necrotic muscle tissue in **3b**-treated group’s thigh portion (Figure 13(d)).

#### Influence of 8b and 3b on VEGF and caspase-3 expression by immunohistochemistry

**Figure 14.**
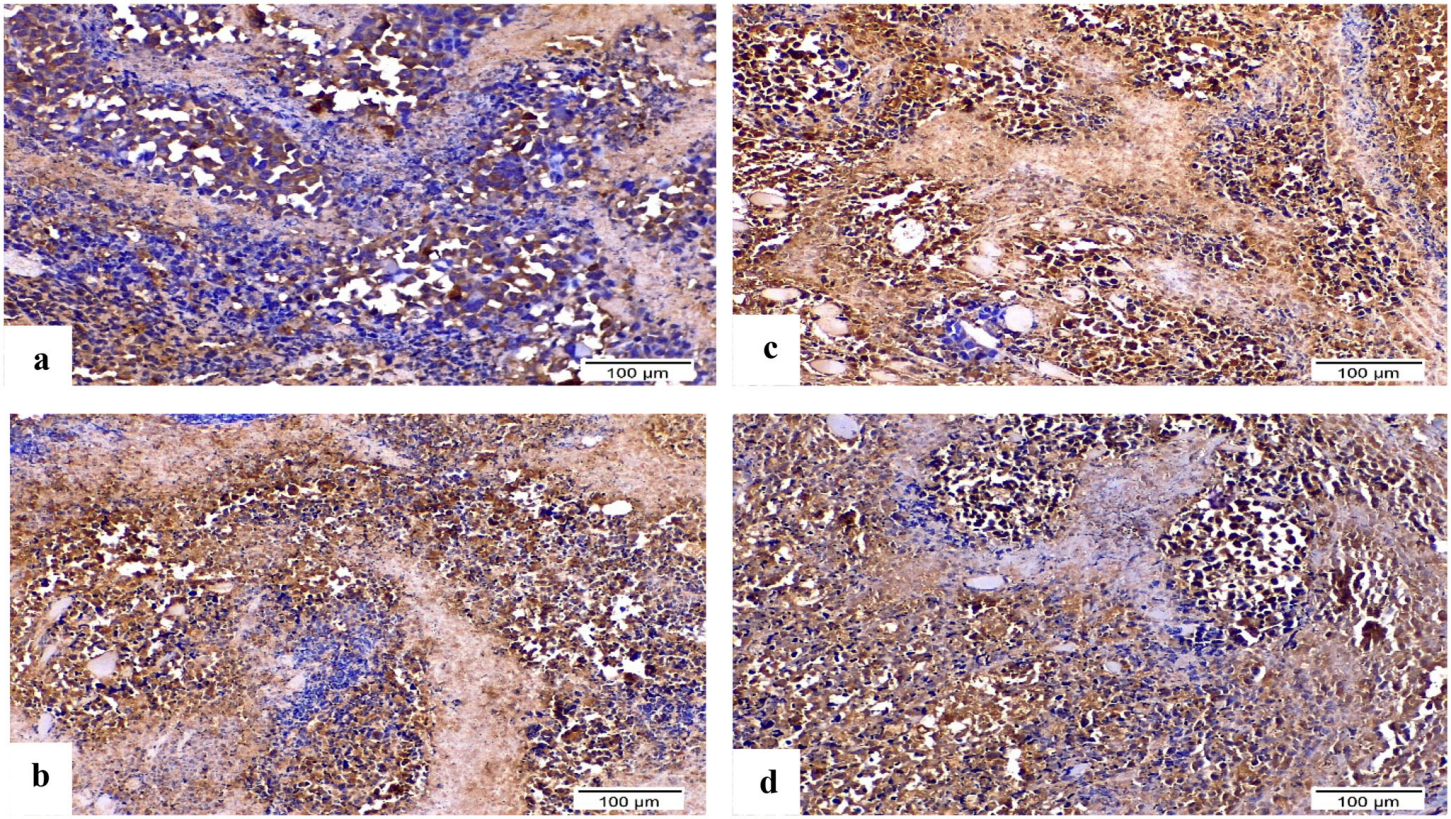
Sections of Ehrlich solid tumour tissue immunohistochemically labelled for caspase-3 (×200). (a) Untreated control lacking detectable antibody for caspase-3. (b) DOX with positive caspase3 immunoreactivity. (c) **8b** exhibited significant positive expression of caspase-3 by the immune system. (d) **3b** displayed positive caspase-3 immunoreactivity.

**Figure 15.**
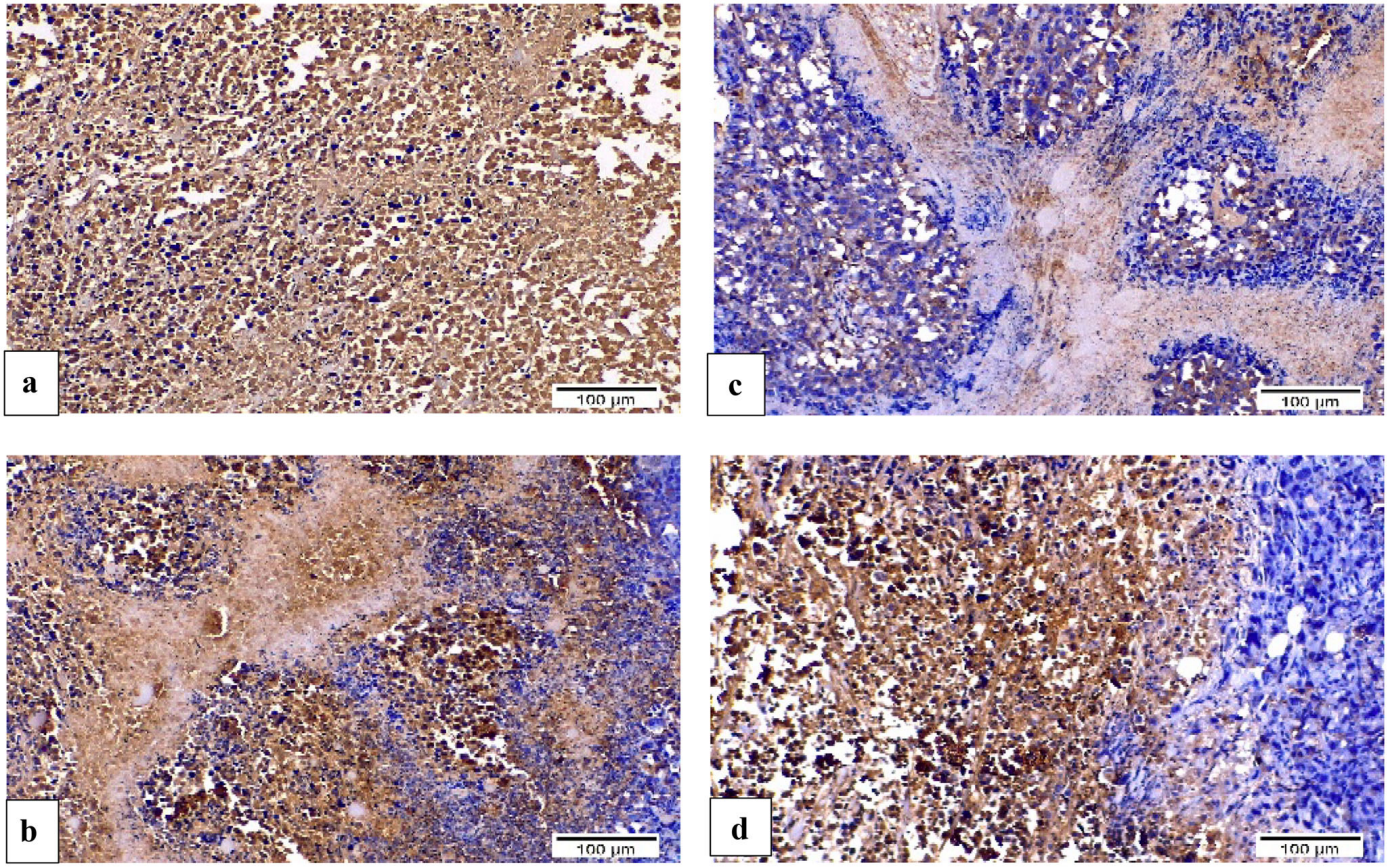
Sections of Ehrlich solid tumour tissue immunohistochemically labelled for VEGF (×200). (a) The untreated control demonstrates high VEGF immunoreactivity. (b) DOX immunoreactivity for VEGF was moderate. (c) **8b** revealed no appreciable VEGF immunoreactivity. (d) **3b** indicated moderate VEGF positivity in the epidermal growth factor receptor.

**Figure 16.**
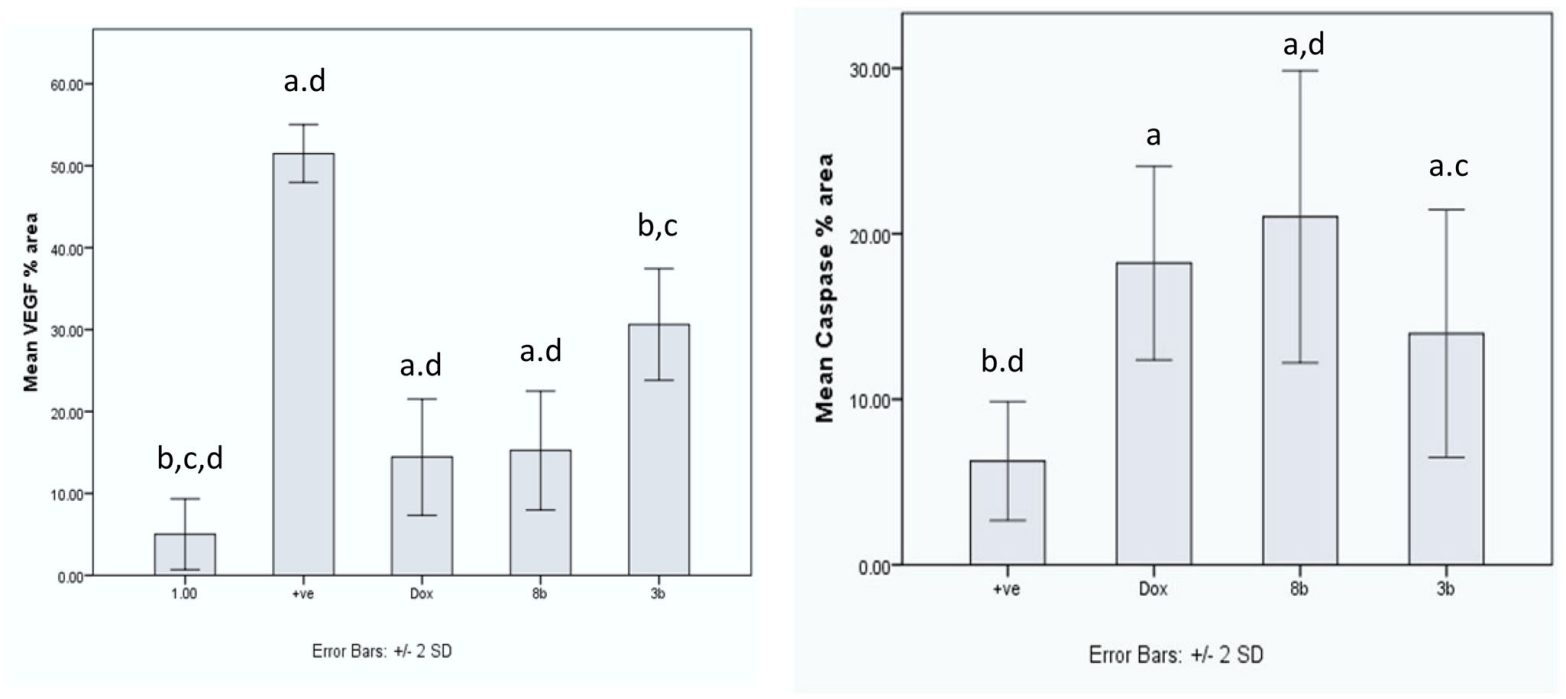
Graphical presentation of VEGF and caspase-3 immunostaining as well as their percentage area in experimental groups. The data were presented as a mean SD (*n* = 6) and analysed using one-way ANOVA followed by Tukey’s post-hoc test with a significance level of *p* = 0.05. Significantly distinct from the positive control group, the DOX group, the **8b** group, and the **3b** group. Significantly distinct from the **8b** group. +ve: untreated control group.

Using immunohistochemistry, robust positive expression of vascular endothelial growth factor VEGF and cleaved caspase-3 were spotted in Ehrlich solid tumour group tissue slices (Figures 14(a), 15(a) and 16). Doxorubicin exhibited moderate caspase-3 expression and low expression of VEGF compared to Ehrlich solid tumour group (*p* < 0.001) (Figures 14(b), 15(b), and 16). Derivative **8b** presented the highest significant increment in caspase-3 expression compared to **3b** (*p* ≤ 0.05) and Ehrlich solid tumour group (*p* < 0.001). Moreover, it exhibited low expression of VEGF compared to Ehrlich solid tumour group (*p* < 0.001) and **3b** with no detected significant difference between **8b** and DOX (Figures 14(c), 15(c), and16). In contrast, derivative **3b** displayed the highest significant increment in the VEGF expression compared with the treated groups with moderate to low expression of VEGF compared to the solid tumour Ehrlich group (*p* < 0.001). Furthermore, it exhibited the lowest caspase-3 expression compared to **8b** group with no significant difference found compared to the DOX group (Figures 14(d), 15(d), and 16).

#### Assay of anti-inflammatory action of 8b and 3b by estimation TNF-α using ELISA assay

**Figure 17.**
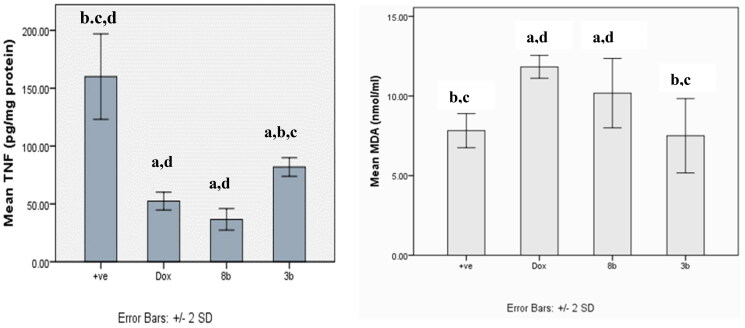
The effect of compounds **8b** and **3b** compared to doxorubicin on levels of TNF-*α* and MDA serum level in in experimental mice. All tested groups were implanted with Ehrlich solid tumour then treated with vehicle only (positive control), DOX, **8b**, or **3b**. The results were shown as mean SD and evaluated using one-way ANOVA followed by Tukey’s post-hoc test at a significance level of *p* = 0.05 (*n* = 6). Significantly distinct from the positive control group, DOX group, **8b** group, and **3b** group. Significantly distinct from the **8b** group.

Ehrlich tumour group showed a statistical significant increment in the release of tumour necrosis factor TNF-*α* compared to other treatment groups (*p* < 0.001). However, there was no statistical significant difference between DOX and **8b**, and no statistical difference was detected between compounds **8b** and **3b** (Figure 17).

#### Assay of lipid peroxidation by estimation MDA by ELISA

The malondialdehyde (MDA) level of all treated groups were significantly higher than those of the untreated control group, with the exception of the **3b** derivative, which did not significantly vary from the untreated control group (Figure 17).

### Normal breast cell line cytotoxicity

The cytotoxicity against human normal breast cell line MCF10A of the promising derivatives **3b** and **8b** were further investigated. Their achieved MCF10A IC_50_ values were 105.33 µM and 53.33 µM, respectively, with selectivity indexes of 18.25 and 5.44, respectively (Table 1). As attained, derivative **3b** showed better safety profile and better selectivity towards the cancerous cells than **8b** and staurosporine. However, both derivatives **3b** and **8b** possessed acceptable safety margin as dictated by M. Suffness who declared that the anticancer agent had acceptable selectivity towards the cancerous cells when its selectivity index exceeds 2^53^ which materialised in both derivatives.

## Conclusion

A variety of substituted bis-thiazole derivatives were designed to preferntially inhibit Pim1 kinase using a calculated pharmacophore model through studying its co-crystallised ligand interaction pattern. They were synthesised in accordance with Hantzsch condensation of substituted α-haloketones and thioamides. The aliphatic **3b** and aromatic **8b** substituted bis-thiazole derivatives showed potent Pim1 kinase inhibition with IC_50_ 0.32 and 0.24 µM, respectively, and breast T47D cytotoxic IC_50_ of 1.46 and 2.61 µM, respectively. Consequently, they inhibited the T47D cell cycle at G0/G1 and S phase where **3b** affected primarily the G0/G1 phase demonstrated 66.5% compared to 53.9% of the control group. On the other hand, **8b** hindered to a greater extent the S phase showing 26.5% compared to 14.9% of the control group. Their potency was rationalised by forming strong H- bonds with the conserved active site Lys67 as revealed from their molecular docking to Pim1 binding site. While the selectivity of **8b** was verified by its binding to the unique Pim1 hinge residue Val126 and its unfavourable Pim2 kinase binding energy. Considering the *in vivo* evaluation, derivative **8b** inhibited the growth of Ehrlich solid tumours in mice by inhibiting the angiogenesis, raising caspase-3, and decreasing the inflammation more than compound **3b**. Consequently, derivative **8b** should be considered a prospective chemotherapeutic agent for further optimisation. Moreover, both **3b** and **8b** showed good selectivity towards the cancerous cells with respective index of 18.25 and 5.44, respectively.

## Supplementary Material

Supplemental Material
