# Supplementary material for: Design, synthesis and cytotoxic evaluation of novel bis-thiazole derivatives as preferential Pim1 kinase inhibitors with in vivo and in silico study
Source: J Enzyme Inhib Med Chem. 2023 Feb 2;38(1):2166936. doi: 10.1080/14756366.2023.2166936 (PMC9897788; doi:10.1080/14756366.2023.2166936)
Supplement: Supplemental Material [file IENZ_A_2166936_SM7300.pdf]

Design, synthesis and cytotoxic evaluation of novel bis-thiazole derivatives as preferential Pim1 kinase inhibitors with *in vivo* and *in silico* study

Mohammad M. Al-Sanea [a], Tamer M. Nasr [b], Samir Bondok [d,e], Aya Gawish [f] and Nada M. Mohamed [b*]

Supplementary Material

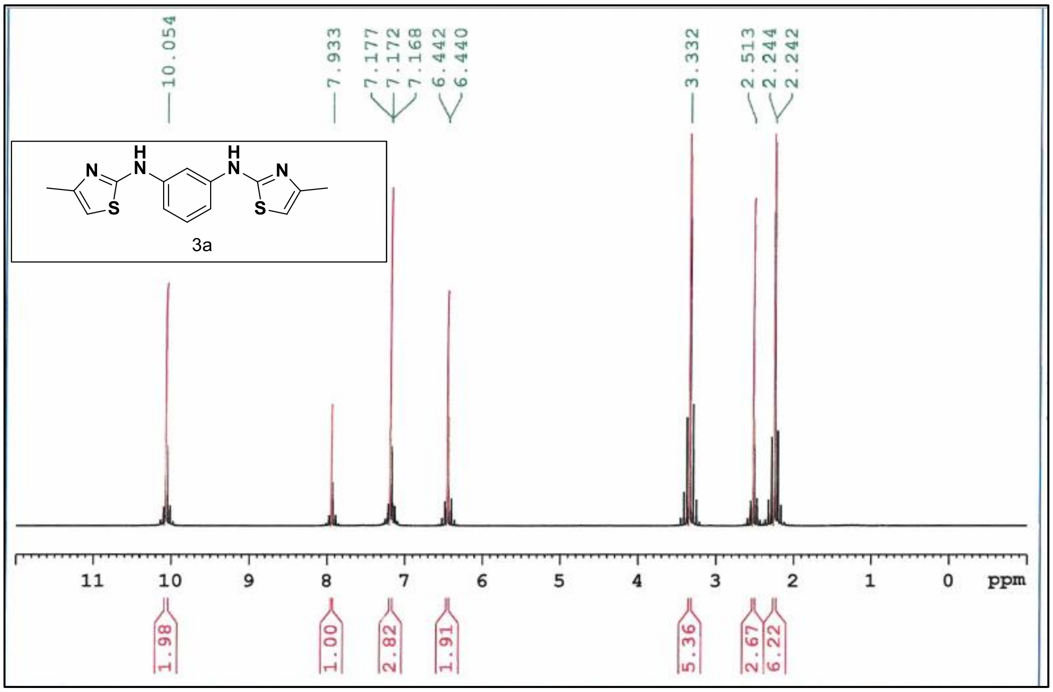

Figure S1. The ^{1}H NMR spectrum (500 MHz, DMSO-d_6) of derivative **3a**.

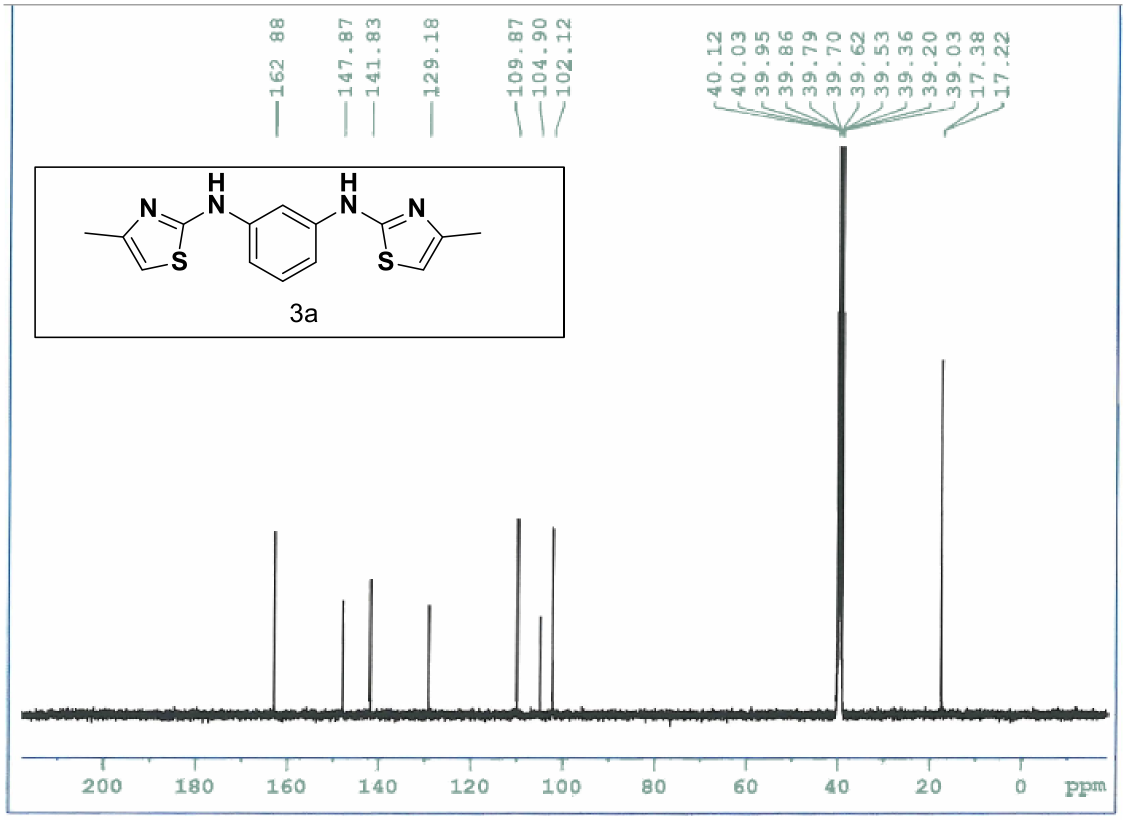

Figure S2. The ^{13}C NMR spectrum of (125 MHz, DMSO-d_6) of derivative **3a**.

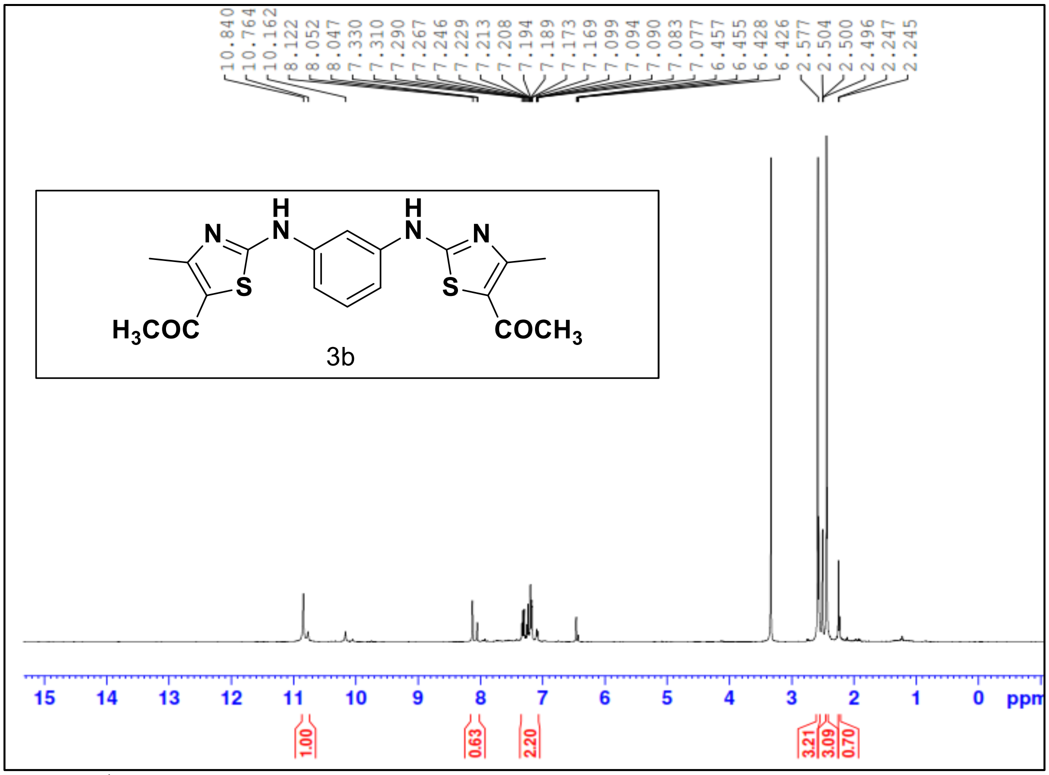

Figure S3. The ^{1}H NMR spectrum (400 MHz, DMSO-d_6) of derivative **3b**.

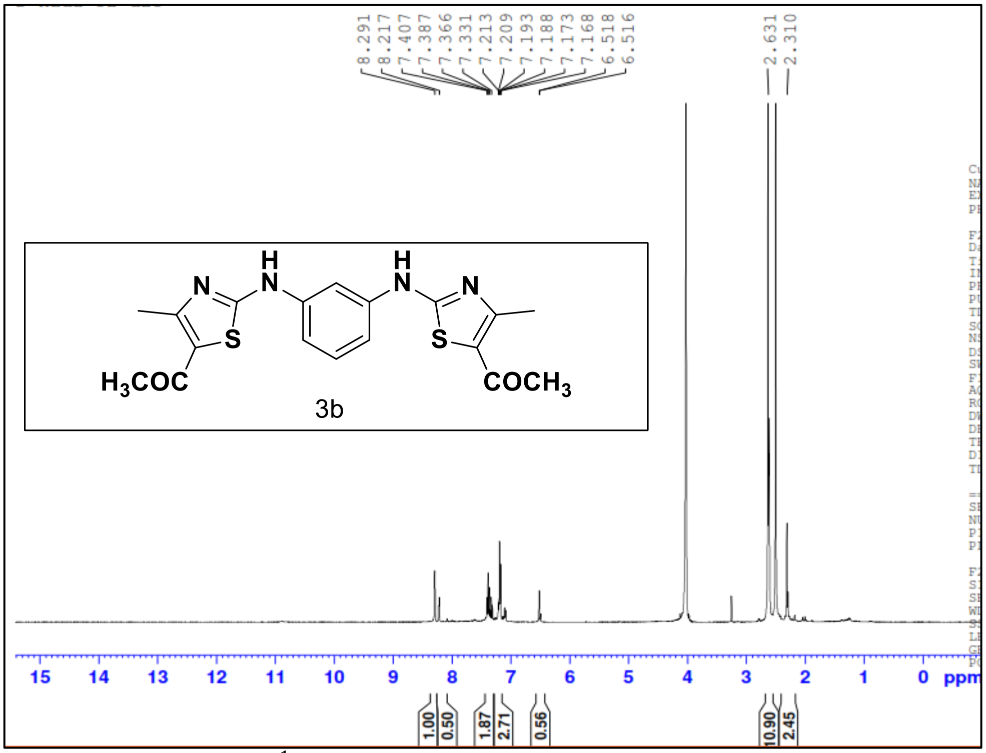

Figure S4. The deuterated ^{1}H NMR spectrum (400 MHz, DMSO-d_6) of derivative **3b.**

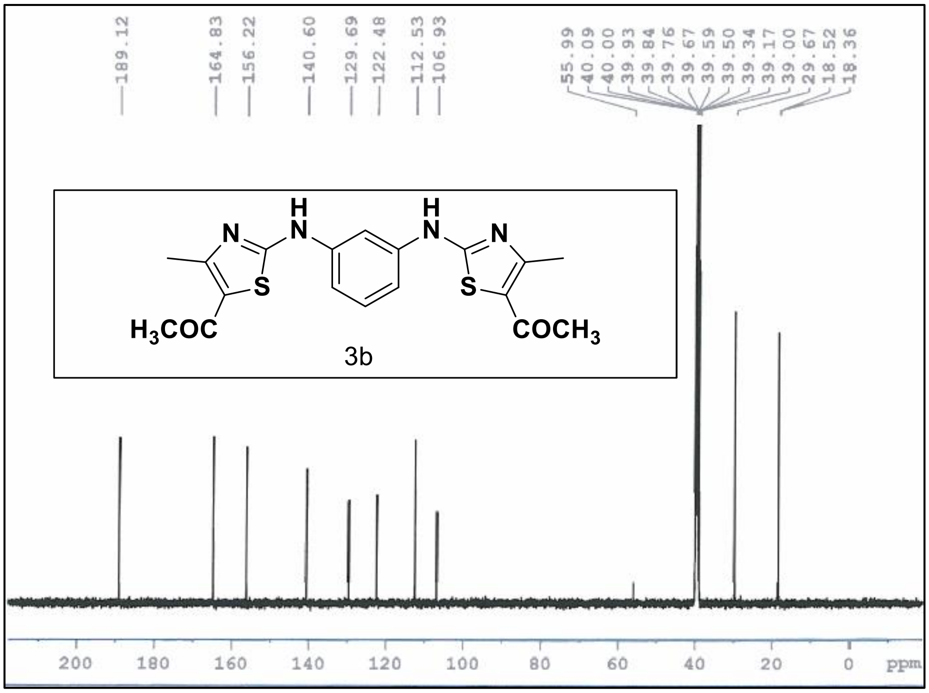

Figure S5. The ^{13}C NMR spectrum (100 MHz, DMSO-d_6) of derivative **3b.**

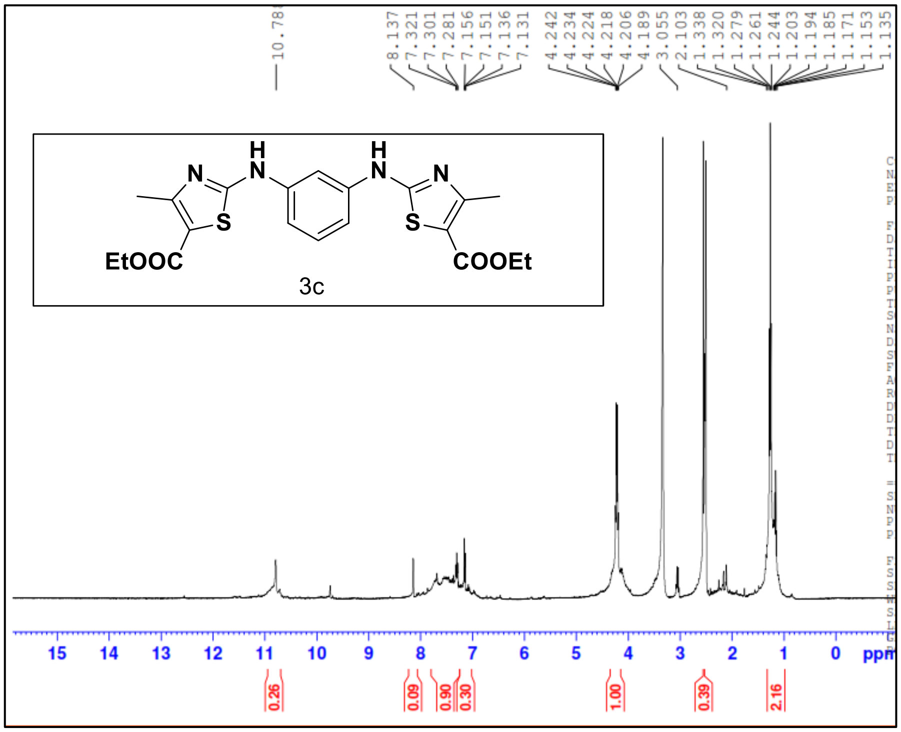

Figure S6. The ^{1}H NMR spectrum (400 MHz, DMSO-d_6) of derivative **3c.**

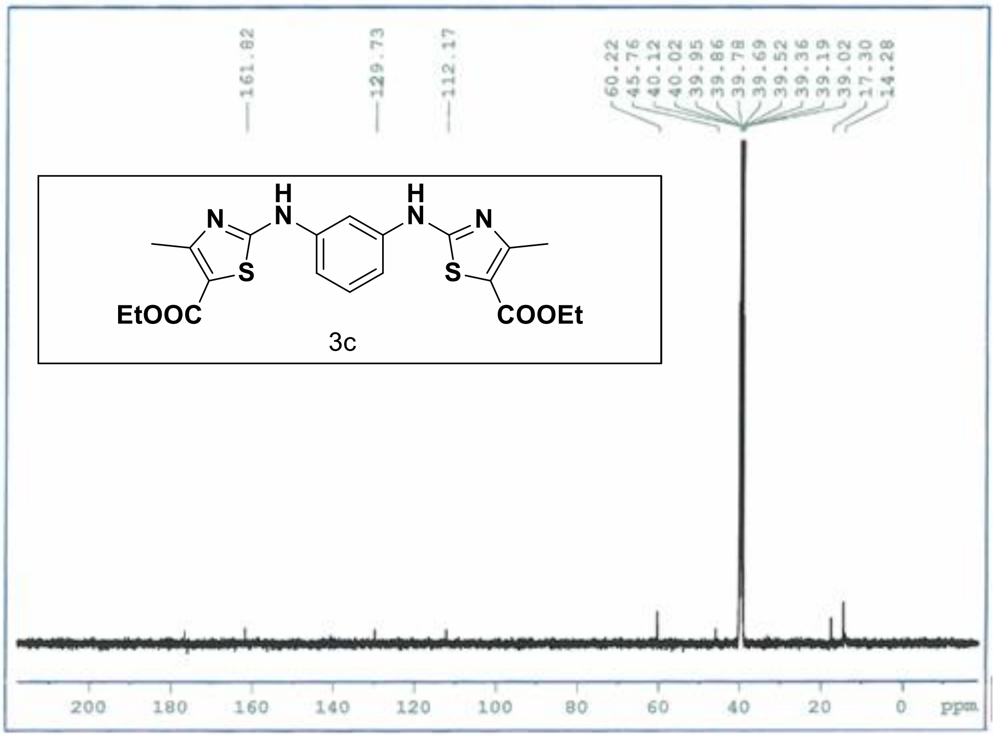

Figure S7. The ^{13}C NMR spectrum (100 MHz, DMSO-d_6) of derivative **3c.**

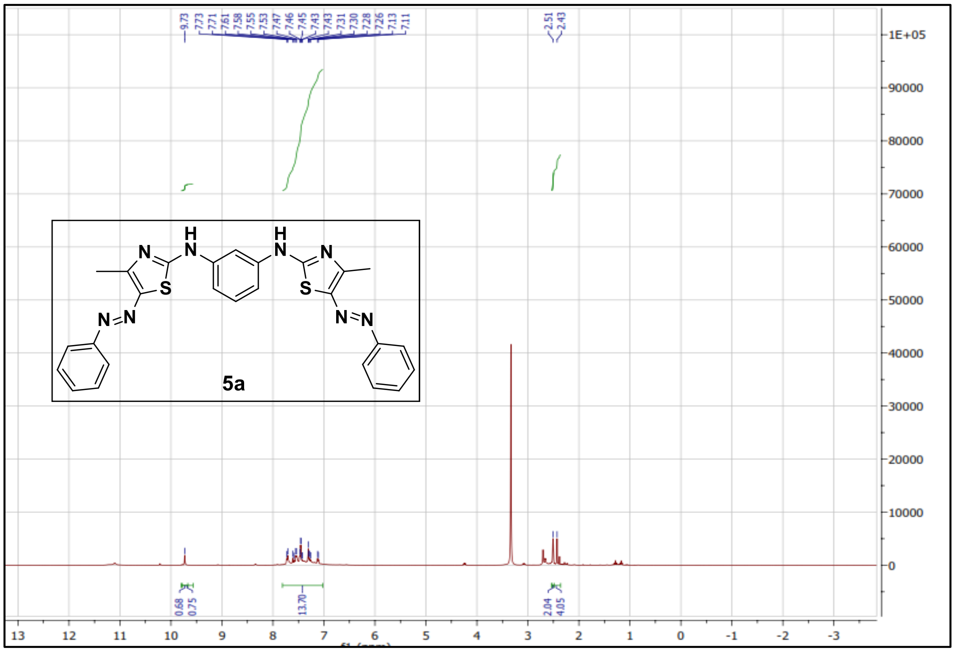

Figure S8. The ^{1}H NMR spectrum (400 MHz, DMSO-d_6) of derivative **5a.**

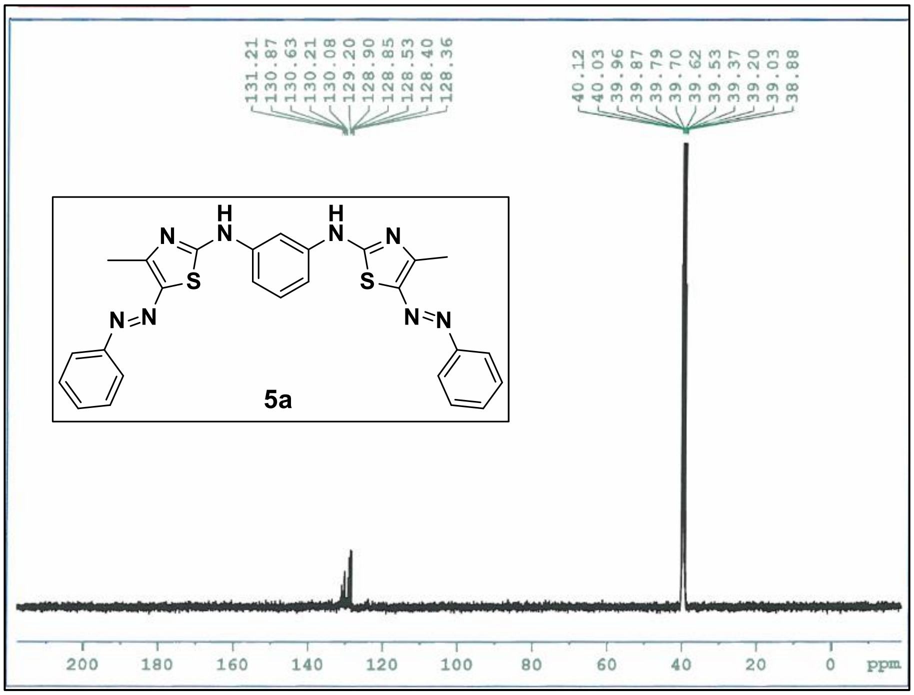

Figure S9. The ^{13}C NMR spectrum (100 MHz, DMSO-d_6) of derivative **5a.**

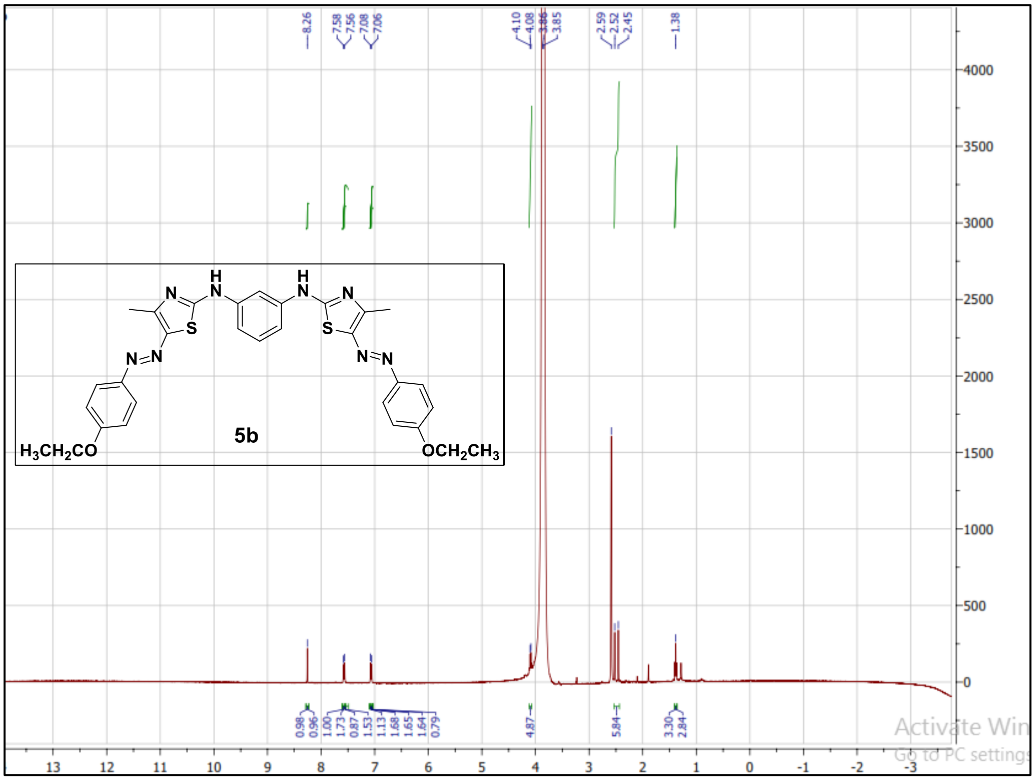

Figure S10. The ^{1}H NMR spectrum (400 MHz, DMSO-d_6) of derivative **5b**.

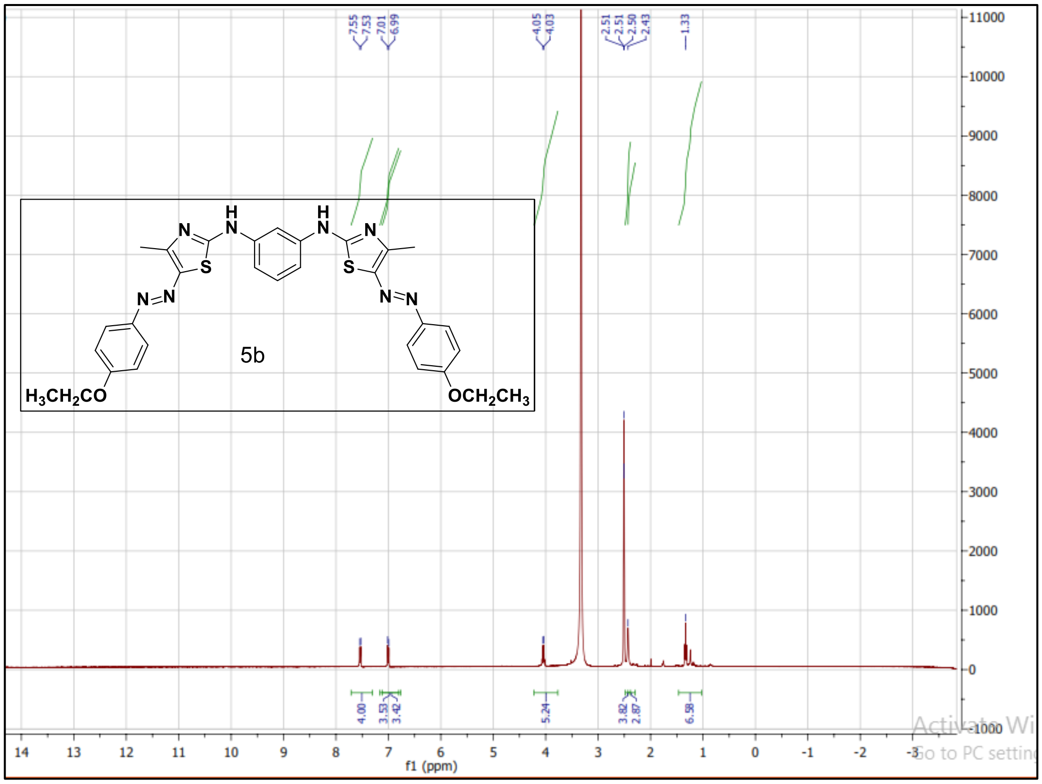

Figure S11. The deuterated ^{1}H NMR spectrum (400 MHz, DMSO-d_6) of derivative **5b.**

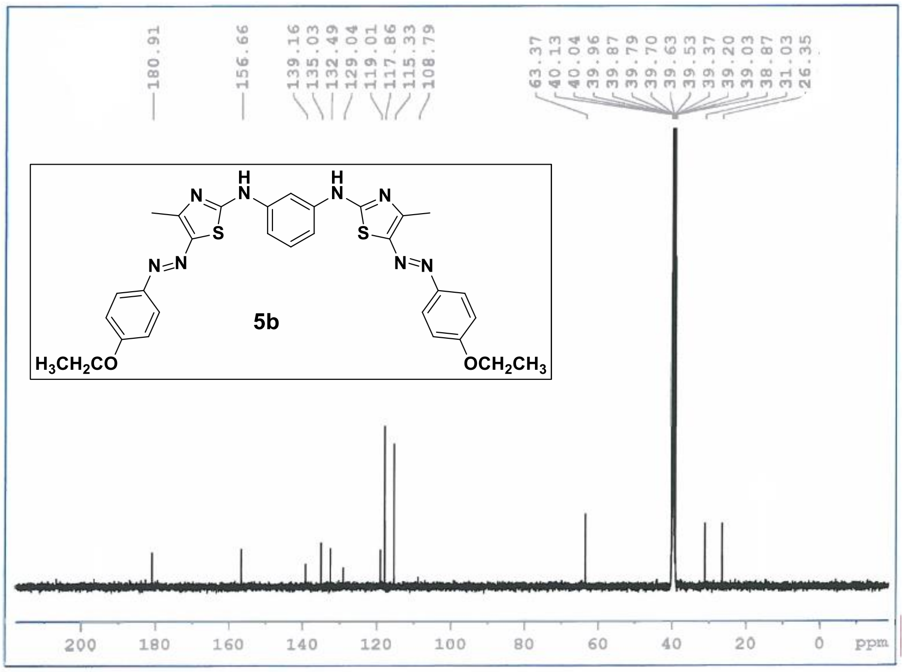

Figure S12. The ^{13}C NMR spectrum (100 MHz, DMSO-d_6) of derivative **5b.**

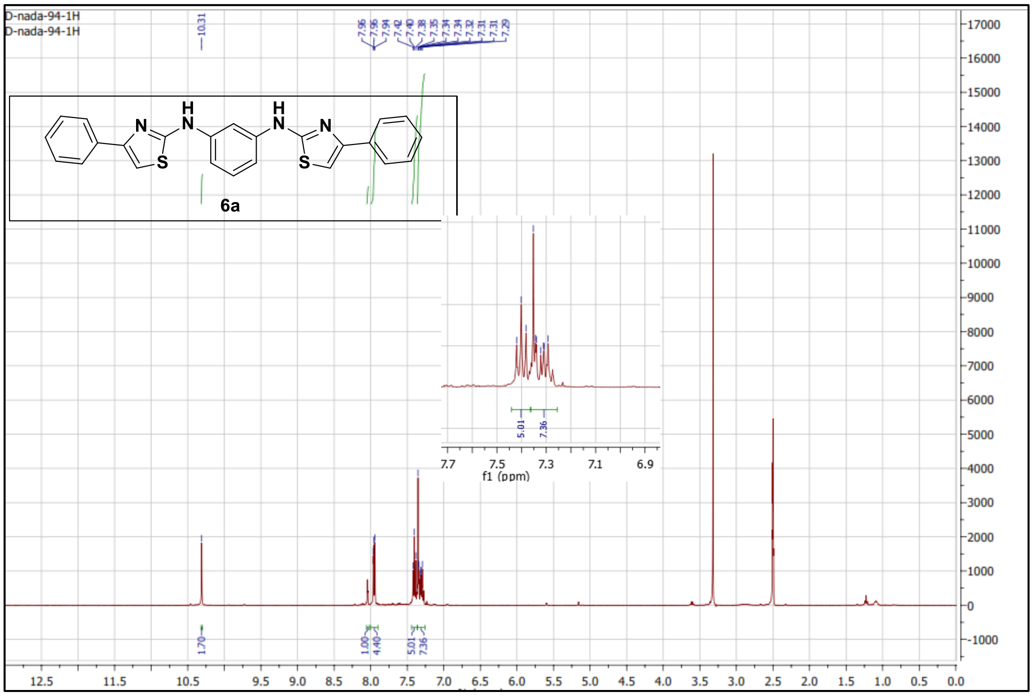

Figure S13. The ^{1}H NMR spectrum (400 MHz, DMSO-d_6) of derivative **6a.**

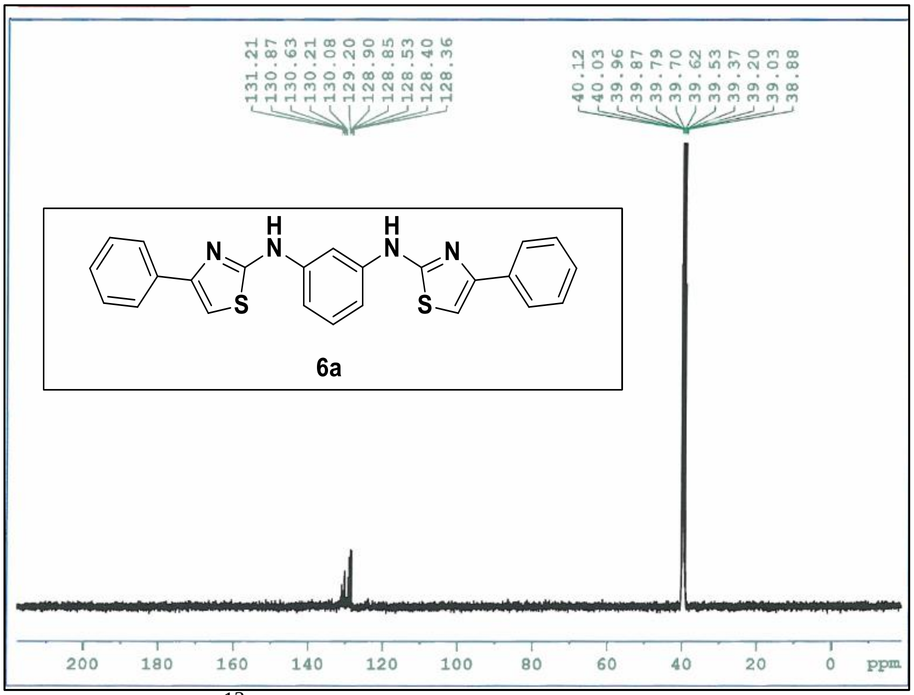

Figure S14. The ^{13}C NMR spectrum (100 MHz, DMSO-d_6) of derivative **6a.**

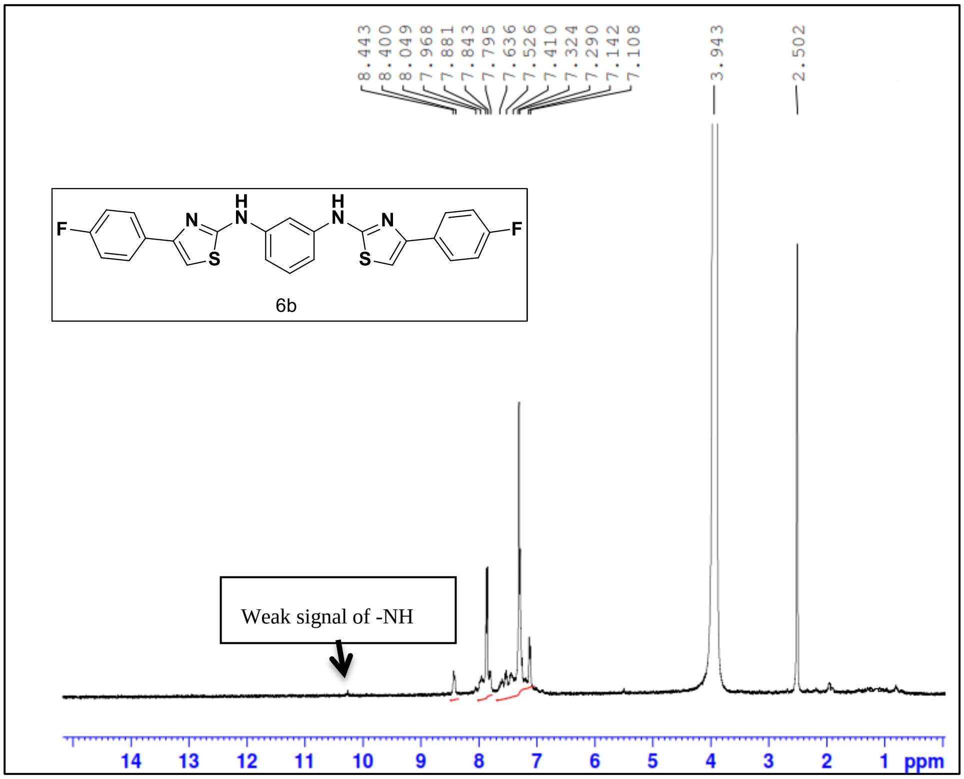

Figure S15. The ^{1}H NMR spectrum (400 MHz, DMSO-d_6) of derivative **6b.**

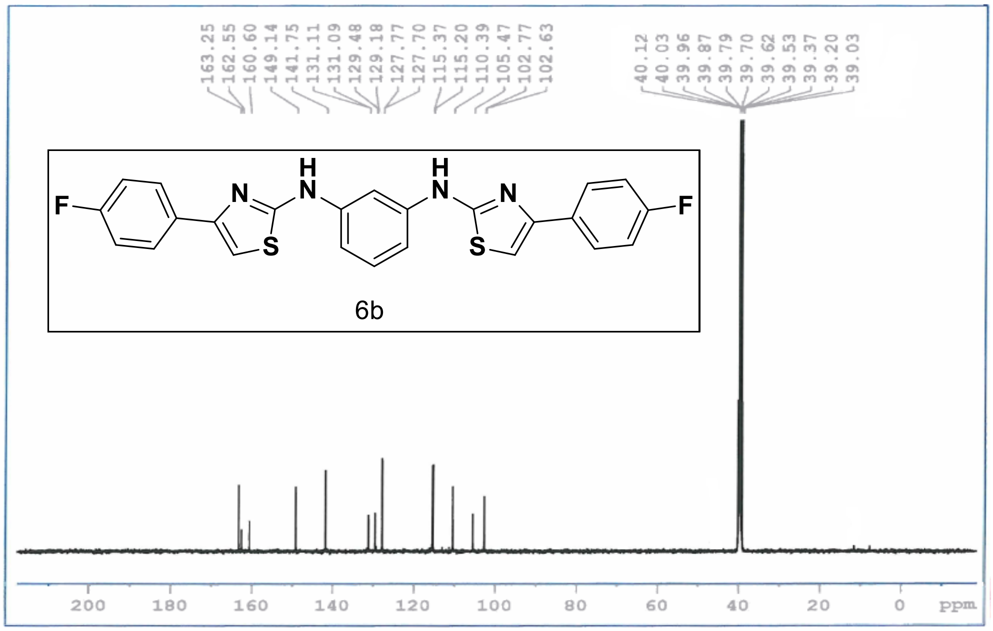

Figure S16. The ^{13}C NMR spectrum (100 MHz, DMSO-d_6) of derivative **6b.**

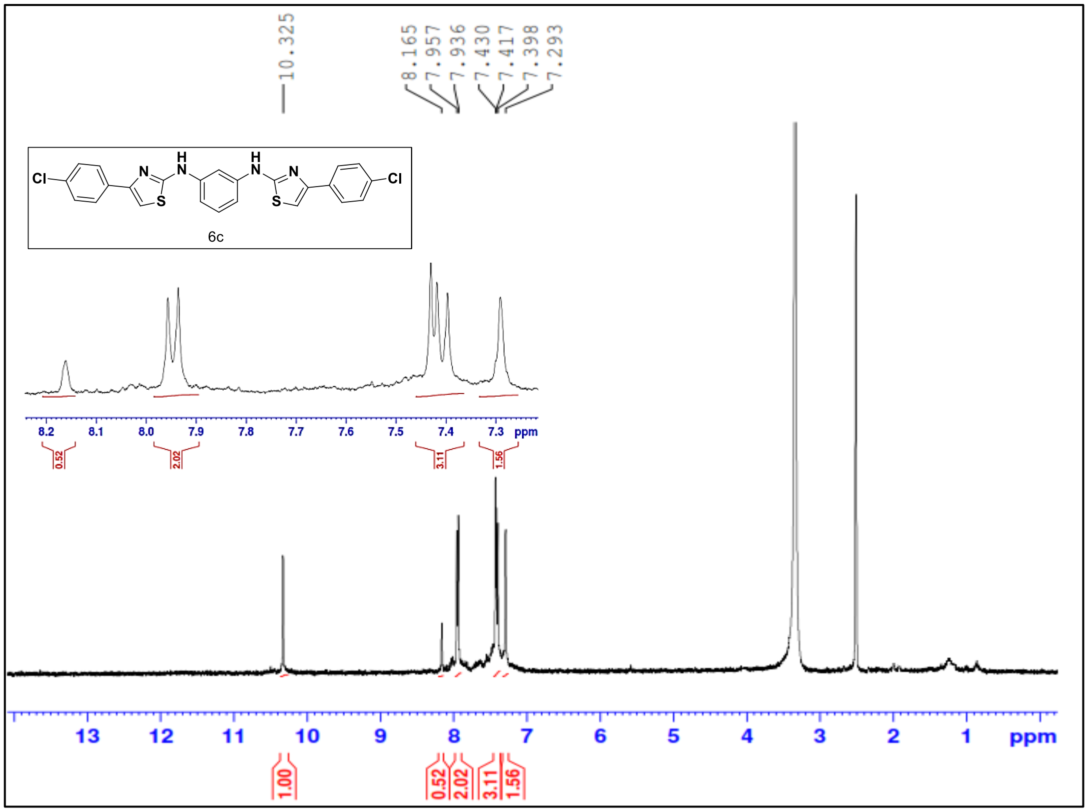

Figure S17. The ^{1}H NMR spectrum (400 MHz, DMSO-d_6) of derivative **6c.**

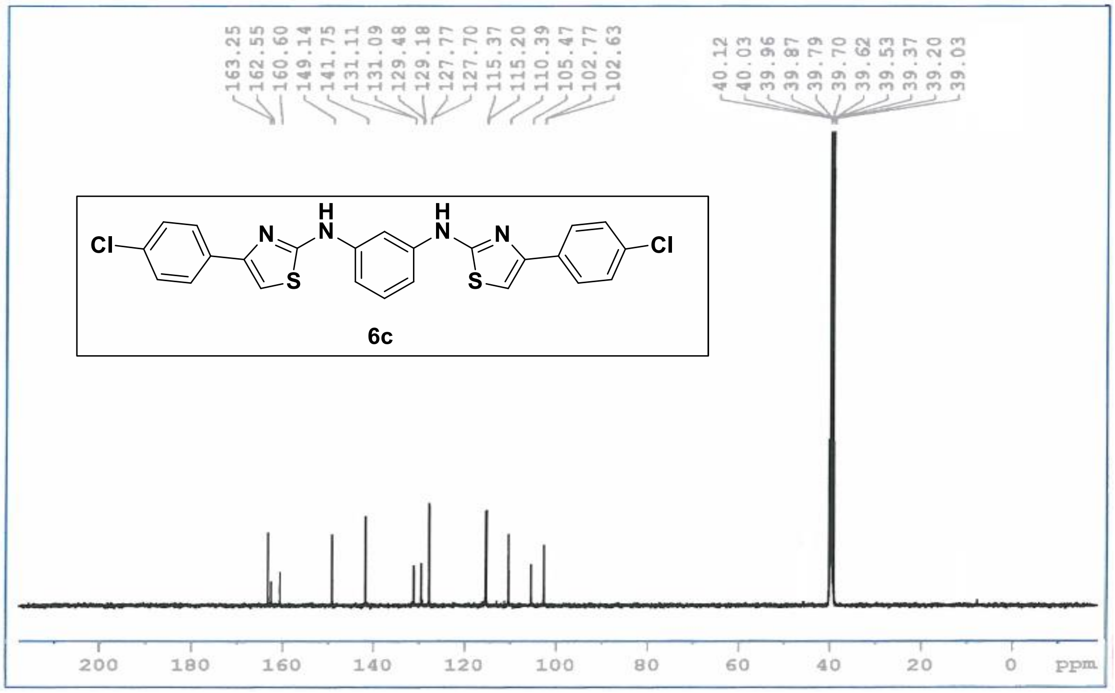

Figure S18. The ^{13}C NMR spectrum (100 MHz, DMSO-d_6) of derivative **6c.**

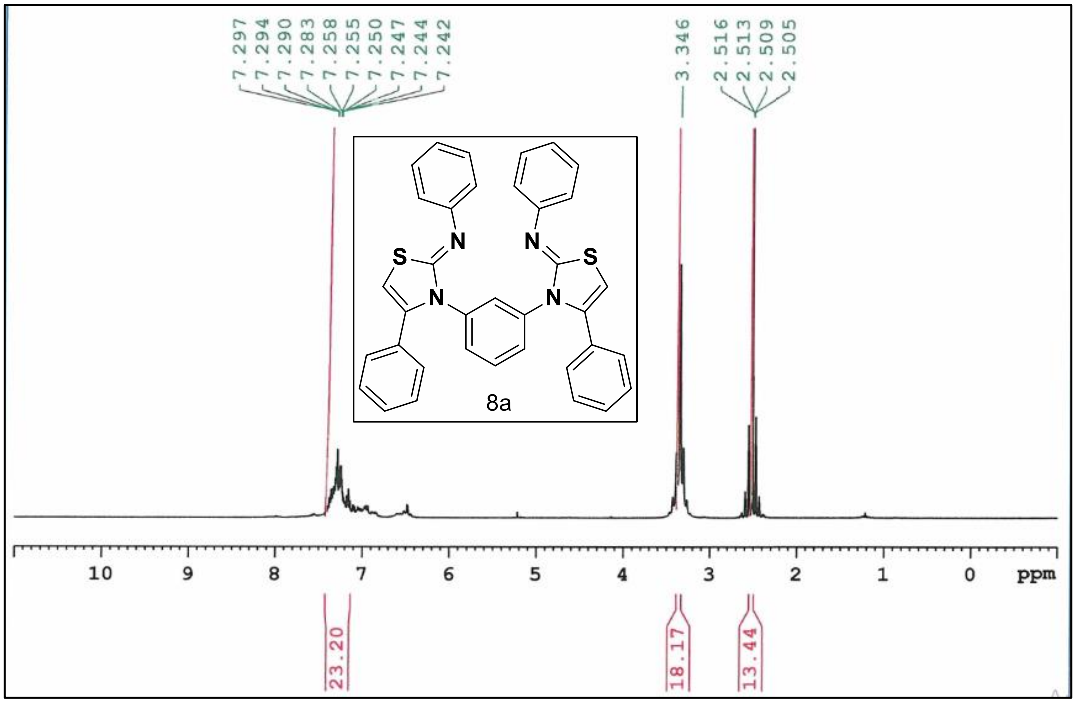

Figure S19. The ^{1}H NMR spectrum (500 MHz, DMSO-d_6) of derivative **8a.**

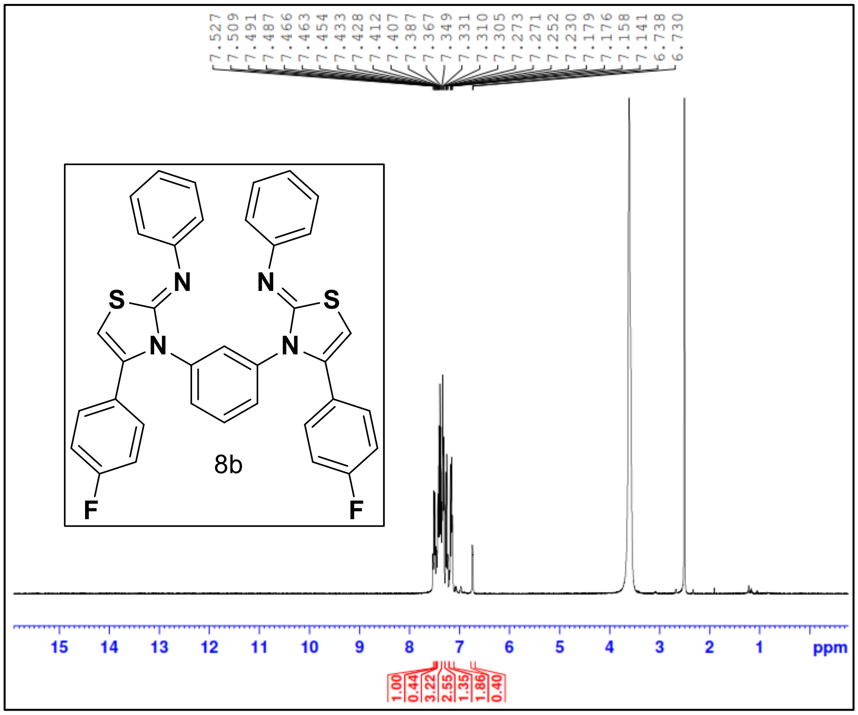

Figure S20. The ^{1}H NMR spectrum (400 MHz, DMSO-d_6) of derivative **8b.**

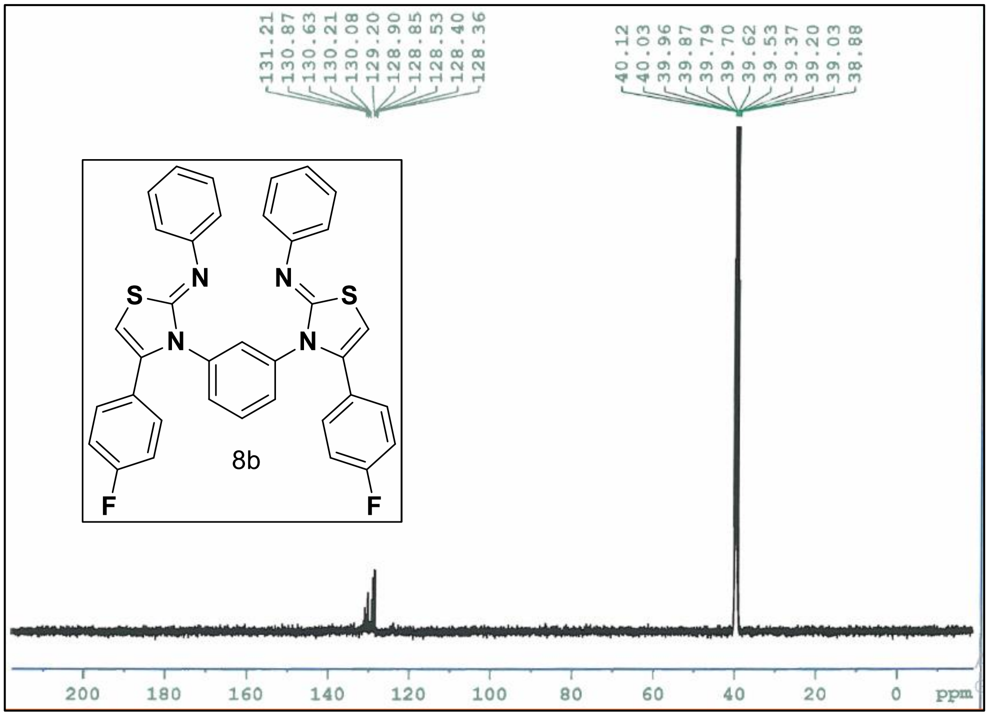

Figure S21. The ^{13}C NMR spectrum (100 MHz, DMSO-d_6) of derivative **8b**.

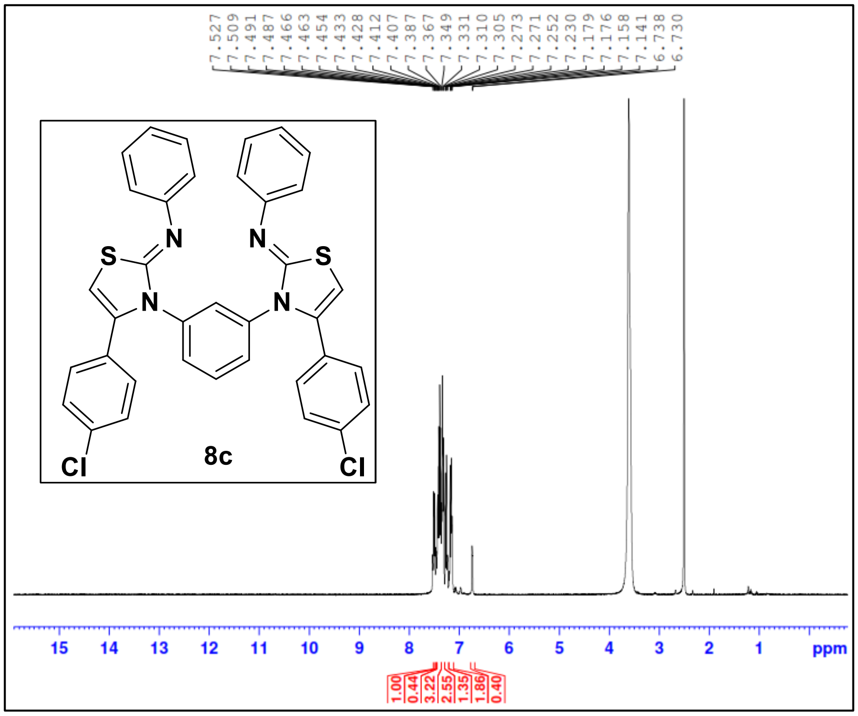

Figure S22. The ^{1}H NMR spectrum (400 MHz, DMSO-d_6) of derivative **8c.**

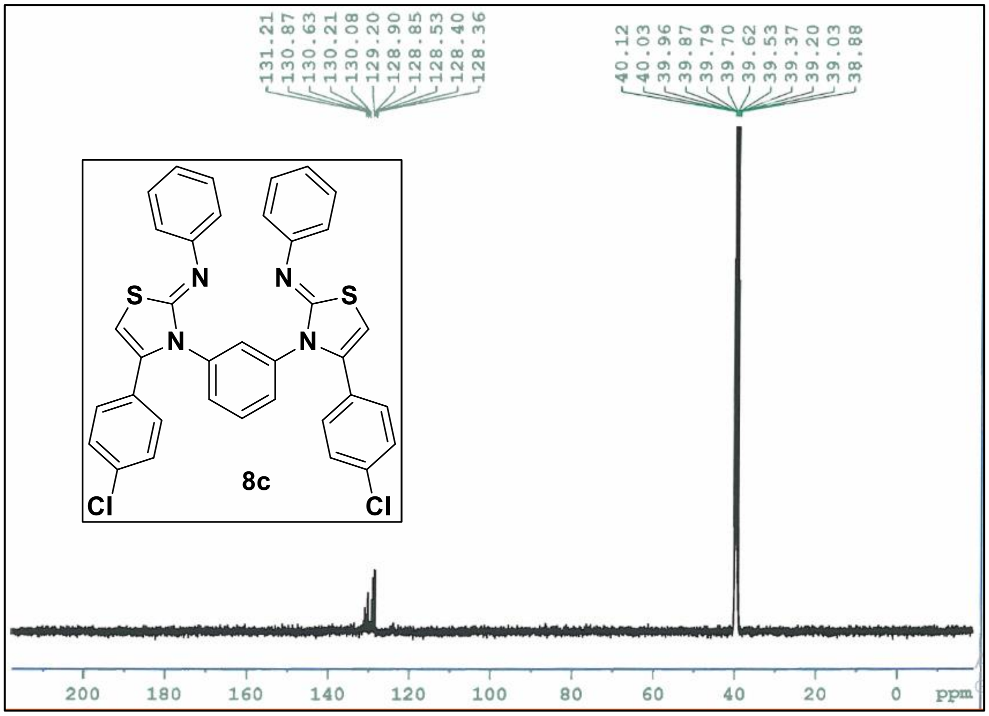

Figure S23. The ^{13}C NMR spectrum (100 MHz, DMSO-d_6) of derivative **8c.**

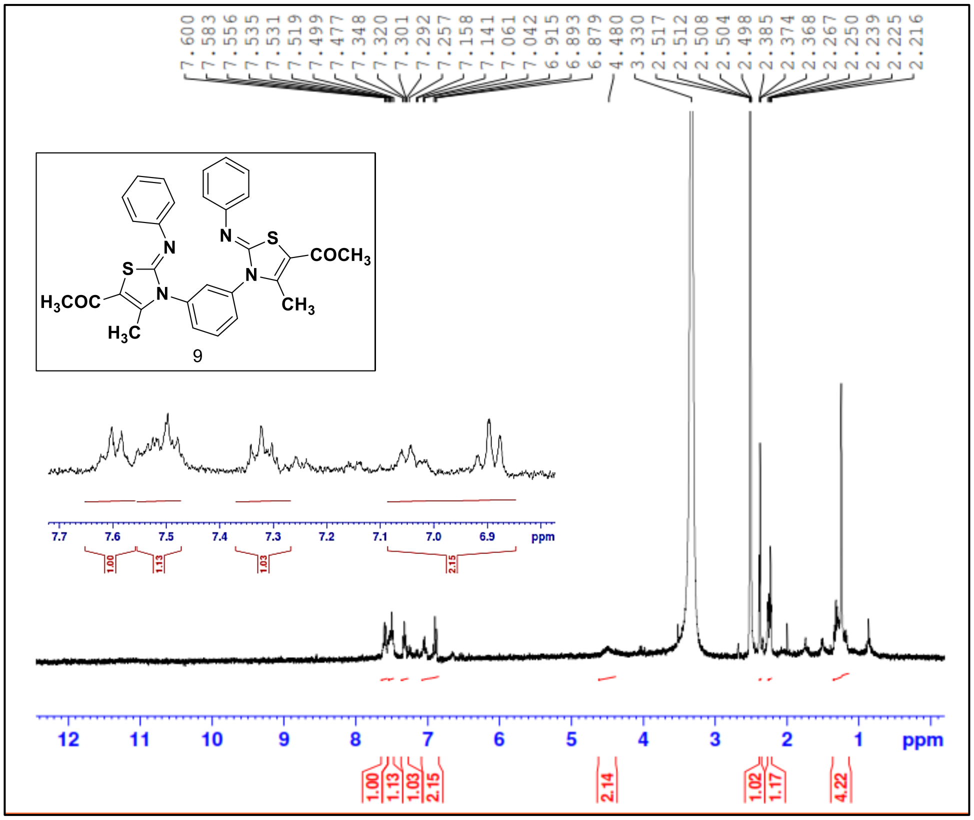

Figure S24. The ^{1}H NMR spectrum (400 MHz, DMSO-d_6) of derivative **9.**

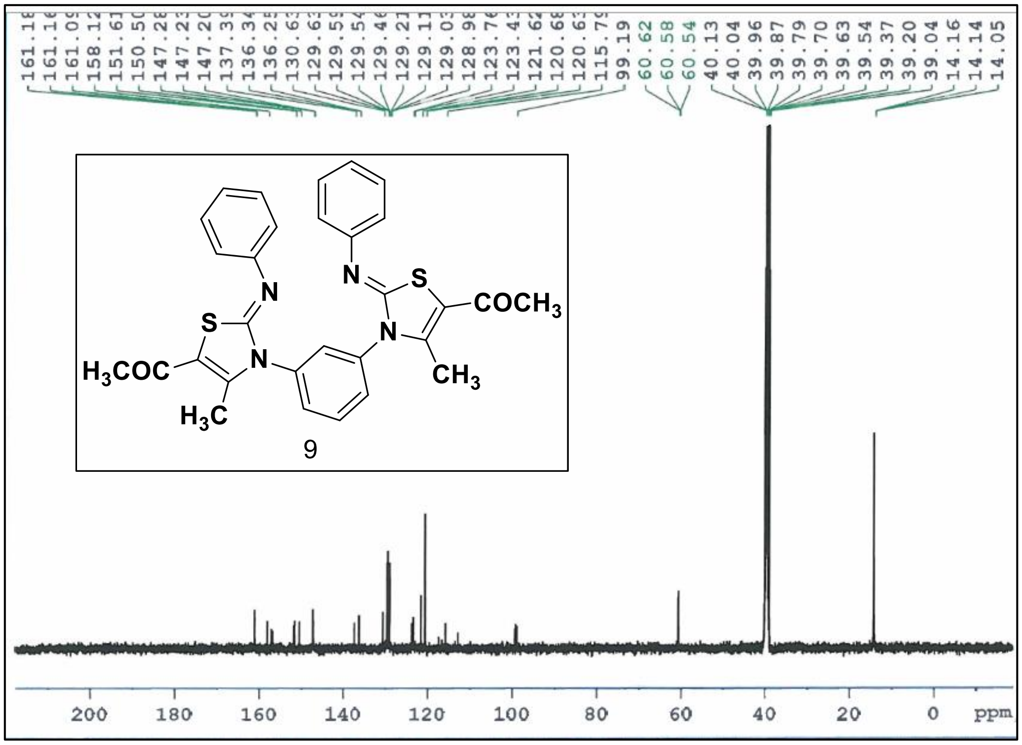

Figure S25. The ^{13}C NMR spectrum (100 MHz, DMSO-d_6) of derivative **9.**

Table S1: The NCI- screening results of derivatives **2-9** over 60 cancerous cell line panel expressed as growth inhibition percent.

Panel name	Cell name	2	3a	3b	3c	5a	5b	6a	6b	6c	7	8a	8b	8c	9
		Percentage of cellular growth inhibition													
Leukemia	CCRF-CEM	3.6	1.5	38.8	11	12.3	-13.9	61.4	40.9	10.4	56.8	-0.9	35.2	32.6	10.4
	HL-60(TB)	0.9	18.2	65.8	22.7	5.5	8.9	34.1	25.7	14.6	-2.5	-4.4	15.8	41.8	14.7
	K-562	90.7	17.1	59.1	39.9	32.9	-0.4	73.4	64	5.8	9.7	7.8	28.8	11.7	5.9
	MOLT-4	10.3	44	61.7	29.2	32.6	-2.2	86.3	53.6	25.4	-5.8	-5.5	50.1	12.3	25.4
	RPMI-8226	-9.5	11.8	32.1	37.7	-9.4	-7.2	50.7	50.5	14.8	-19.2	-0.5	24.2	37	14.8
	SR	NT*	17.3	61.5	36.1	NT	-15.5	NT	48.6	3.9	NT	6.1	36.1	12.1	3.9
Non-Small Cell Lung Cancer	A549/ATCC	6.1	-3.9	34.2	12	36.4	12.5	55.8	37.2	6.7	0.1	-4.9	19.8	27.7	6.7
	EKVX	-1.3	14.7	31.2	38.6	16.9	6.5	62.3	60.2	12.4	2.5	5.3	19.5	4.9	12.4
	HOP-62	-19.5	3.5	22.2	1.2	9.3	-1.3	27	27.3	17.7	-18.9	-12.6	-4.6	-3.3	17.7
	HOP-92	-4.5	41.2	36.4	47.8	-4	25.1	56.3	60.8	59.5	-15.7	14.5	22	2.4	59.5
	NCI-H226	1.9	24.2	34.6	15	33	14.8	33.8	48.5	27.2	-6.2	17	23.5	16.9	27.2
	NCI-H23	1.5	21.2	36.8	41.6	14.7	10.6	63.2	NT	10.1	-5.7	NT	NT	NT	10.1
	NCI-H322M	11.7	4.9	38.3	3.8	22.8	11.4	41.9	39.9	5	2.6	1.8	15.2	-1.2	5
	NCI-H460	-9.7	1.6	54.4	23.5	3.2	-6.8	73.1	59.6	-2.1	-16.5	-6.3	37.5	0.1	-2.1
	NCI-H522	2.8	39.3	53.8	26.7	30.7	12.9	88.6	93.8	13.2	-6.3	12.2	41.6	42.2	13.2
Colon Cancer	COLO 205	-36.7	-3.8	22.3	12.8	-4.9	-5.1	16.5	24.3	-7.3	-37.6	-21.3	-0.4	17.2	-7.3
	HCC-2998	-16.9	-4.2	19.9	9.7	-0.4	-4.6	64.5	64.8	-10.9	-11.5	-22.2	12.1	-1.7	-10.9
	HCT-116	-7	4.8	55.8	43.5	14.1	3.8	60	42.3	38.5	-11.8	-4.1	16.7	15.7	38.5
	HCT-15	4.9	7.3	55.1	20.9	21	8.4	69.5	47.5	7.1	0.8	-4.7	21.7	-1.3	7.1
	HT29	-13.3	-6.8	30	-4.7	2	0.2	60.6	37.7	-10.5	-26.3	-3.1	9.9	12.4	-10.5
	KM12	12.4	5.2	39.8	12.2	52.1	-0.8	98.9	62.4	-1.4	1	1.1	12.8	8.8	-1.4
	SW-620	-6.9	-3.6	37.5	11.4	9.4	-0.3	33.8	29.7	0.4	-16.8	-0.4	45.1	36.7	0.4
CNS Cancer	SF-268	9.1	1.4	28.9	14.8	42.4	4.5	59.4	42.1	22.6	3.3	0.3	8.7	12.1	22.6
	SF-295	9.8	9.5	13.5	33.8	13.6	4.4	62.4	9.6	4	1.2	-22.4	-18.8	-17.9	3.9
	SF-539	-0.5	7.9	27.8	16.2	15.4	0.1	42.3	29.1	13.9	-11.5	-7.1	-0.9	-3	13.9
	SNB-19	11.7	1	9.1	17.7	42.6	11.5	42	29.8	11.5	3.4	-5.9	5.6	3.8	11.5

	SNB-75	2.4	1.6	37.1	2.6	38.4	7.8	51.4	4.8	22.5	7.9	11.7	15.4	14.6	22.5
	U251	11.2	3.5	34.3	16.7	30.1	3.3	48.8	37.3	2.2	-1.4	2.7	28.1	11.1	2.2
**Melanoma**	LOX IMVI	NT	25.5	51.9	25.5	NT	2.6	NT	60.7	7.3	NT	22.6	23.9	50.6	7.2
	MALME-3M	15.7	-18.3	30.2	15.2	-7.7	-6.9	47.2	17	-22.4	10.8	3	7.5	6.7	-22.4
	M14	-25.1	-7.3	48.7	14	-7.6	-7	52.2	33.2	29.3	-19.6	-14.3	0.4	-10.7	29.3
	MDA-MB-435	2.7	4	40.9	17.9	7.9	1.6	55.3	36.9	-7.1	-2.8	1	13.1	5.4	-7.1
	SK-MEL-2	-11.7	NT	NT	NT	-6.7	NT	35.1	65.3	NT	-35.3	12.6	22.2	-9.6	NT
	SK-MEL-28	-3.8	10.4	28.5	15.7	2.5	2.5	39.1	23.6	-4.2	-9.8	-3.6	1.7	-1	-4.2
	SK-MEL-5	8.6	6.2	44.2	55.1	8.5	0.8	79	38.8	2.9	1.5	4	9.3	3.1	2.9
	UACC-257	1.9	-21.6	11.3	10.3	2.7	-9.2	53.9	25.4	-20.4	-1.7	-11.8	-7	-8.4	-20.4
	UACC-62	29.5	22.3	51.2	33.1	30.9	20.5	49.7	42.9	17.4	5.8	20.3	41	10.5	17.4
**Ovarian Cancer**	IGROV1	2.5	7.9	73.6	23.4	52.2	9.7	65.9	58.2	9	-3.3	-6.1	12.9	-0.4	8.9
	OVCAR-3	11.5	NT	NT	NT	26	NT	46.8	42.5	NT	-6.1	-3.6	4.1	-6.6	NT
	OVCAR-4	5.4	11.8	48.5	33.2	30.8	0.1	62.4	44.3	8.4	-6.6	5.3	34.7	12	8.4
	OVCAR-5	-3.6	7.1	16.5	8	19.8	9	35.5	34.5	9.6	-4.9	-2.9	14	-2.6	9.6
	OVCAR-8	6	21.2	56.6	17.3	33.4	-4	60.1	32.1	7.7	4	-1.5	12.4	7.9	7.7
	NCI/ADR-RES	2.4	20.1	44.4	33.1	10.2	1.5	73.9	36.3	4.6	-9.3	-5	10.2	0.5	4.5
	SK-OV-3	-25.8	10.7	74.2	1.3	-6.6	1.1	2.5	13.2	8.3	-29	-34.8	-15.4	-29.4	8.3
**Renal Cancer**	786-0	-7.8	-10.8	44.3	5.5	18.7	6.6	49.6	31.2	29.2	-13.4	-11.1	4.2	-6.9	29.2
	A498	-1.2	51.2	72.3	52.7	1.2	13.5	14.5	21.7	9.8	-17.7	-18.1	-6.4	-16.3	9.8
	ACHN	3.7	20.7	63.5	24.6	38.3	1.1	63.8	48.2	14.3	-3.8	3	2.3	-1.5	14.3
	CAKI-1	20.3	23.7	71.9	21.9	54.8	8.3	65.1	53.6	43	10.9	25.4	22.2	9	42.9
	RXF 393	-3.8	4.8	53	9	-4.4	1	36.2	24.5	16.7	-16.6	10.3	11.6	8.4	16.7
	SN12C	18.1	13.8	45	11.8	53.9	4.7	56.6	52.8	1	4.5	3.9	7.5	2.3	0.99
	TK-10	-7.3	-35	46.4	-0.8	2.9	-0.2	31.4	5.5	1.4	-17.5	-38.8	-16.6	-14.1	1.4
	UO-31	30	15.7	67.8	32.9	69.7	12.4	67.7	68.7	21.6	14.6	25.4	26.3	17.8	21.6
**Prostate Cancer**	PC-3	-2.4	20.2	48.7	22.9	-3.3	6.2	67.9	64.8	22.2	-24	21.8	32.1	28.9	22.2
	DU-145	-1.2	10.2	54.6	16.2	16.6	-3.9	41.1	32.3	-1.8	-10.2	-3.2	17.1	19.2	-1.8
**Breast Cancer**	MCF7	8.3	21.9	38.3	26.7	52.4	10.6	84.4	75.3	9.8	8.5	18	42.9	23.4	9.8
	MDA-MB-	10.3	21.2	19.3	20.8	35.1	6.7	52	35.4	24.9	-0.2	-4.1	8.9	-4.5	24.9

	231/ATCC														
	HS 578T	-5	7.9	47.3	14.7	19.8	2.2	43.5	30.7	29.5	3.9	4.2	13.4	10.4	29.5
	BT-549	-14.6	-24.4	53.8	26.3	5.8	-3.7	68.3	36	-6.1	-13.2	-31.8	-4.1	-15.5	-6.1
	T-47D	13.2	36.8	86.7	63.9	48	17.1	76	92.6	18.3	6.3	10.2	51	8.9	18.3
	MDA-MB-468	13.5	15.9	45	52.4	29.4	4.3	60.8	61.4	13.4	-8.4	9	51.2	35.4	13.5
